# The correlation between Diabetes and age-related degeneration and the static and dynamic 3D mechanical distribution of different plantar regions

**DOI:** 10.3389/fendo.2024.1433928

**Published:** 2024-11-25

**Authors:** Xiong-gang Yang, Xing-xi Hu, Qi-yang Wang, Zhi Peng, Hao-tian Luo, Sheng Lu

**Affiliations:** ^1^ Department of Orthopedics, The First People’s Hospital of Yunnan Province, The Affiliated Hospital of Kunming University of Science and Technology, Xishan District, Kunming Yunnan, China; ^2^ The Key Laboratory of Digital Orthopedics of Yunnan Province, Xishan District, Kunming Yunnan, China; ^3^ Department of Orthopedic and Trauma Surgery, The Affiliated Hospital of Yunnan University, Kunming, China

**Keywords:** aging, Diabetic foot, mechanical distribution, plantar pressure, plantar shear force

## Abstract

**Purpose:**

This study aimed to compare the distribution of plantar pressure and anterior-posterior (AP) or medial-lateral (ML) shear forces in healthy younger (HY) people, healthy older (HO) people, and diabetic patients, both in static standing and during gait.

**Materials and methods:**

A total of 20 HY adults, 16 HO adults and 15 diabetic patients were included. The static mechanical distribution measurements included: static horizontal, AP slope plane, and left/right slope standing. Data collected during the gait cycle encompassed the plantar pressure-time integral (PTI), peak pressure (PP), AP/ML shear force-time integral (AP-STI/ML-STI), and AP/ML peak shear force (AP-PS/ML-PS). The plantar surface was segmented into regions including hallux (HL), 2^nd^~5^th^ toes (T_2-5_), 1^st^ metatarsal head (M_1_), 2^nd^~3^rd^ metatarsal heads (M_2-3_), 4^th^~5^th^ metatarsal heads (M_4-5_), lateral foot arch (LA), and heel regions.

**Results:**

The HO group exhibited increased static pressure in M_2-3_ and heel regions and AP shear force in the entire plantar and M_1_ regions, in comparison to the HY group. The diabetes group showed increased static pressure in entire plantar, M_1_, M_2-3_ and heel regions and AP shear force in the entire plantar, T_2-5_, M_1_, M_2-3_ and heel regions. During gait, the HO group exhibited increased PTI in the whole plantar, T_2-5_, M_2-3_, and M_4-5_ regions, while the diabetes group showed increased PTI in the whole plantar, M_1_ and M_2-3_ regions. The HO group showed increased PP in the whole plantar, M_1_ and heel regions, while decreased in the M_2-3_ region. The diabetes group showed increased PP in the whole plantar, T_2-5_, M_2-3_, M_4-5_ and heel regions. The HO group showed increased AP-STI in the T_2-5_, M_1_, and M_2-3_ regions, while the diabetes group showed increased AP-STI in the whole plantar, M_2-3_ and heel regions.

**Conclusions:**

Our findings indicate that both static and dynamic plantar pressures and shear forces are significantly greater in diabetic patients and HO individuals compared to HY adults. The most substantial increases was occurred under the M_2-3_ and heel regions.

## 1 Introduction

With the continuous improvement of living standards and the aging population, the number of diabetes patients is continuing to rise (1, 2). Among the various complications associated with this chronic condition, diabetic foot stands out as one of the most frequently encountered, affecting an estimated 15-25% of individuals with diabetes (3). The treatment outcomes for diabetic foot ulcer (DFU) are not satisfactory, with the majority of patients eventually undergoing lower limb amputation within four years of diagnosis (3). Research indicates that up to one-third of the healthcare expenditures for diabetes management are attributable to the treatment of DFU (4, 5). Evidence further suggests that with early multidisciplinary care, nearly 50% of amputations in DFU patients can be avoided (6). This finding underscores the growing importance of investigating strategies for proactive ulcer prevention, which could substantially mitigate the clinical and economic burden associated with DFU.

Hyperkeratosis and callus formation on the plantar foot are important risk factors for DFU (7, 8). The main cause of plantar callus formation is increased plantar pressure and shear forces. Numerous studies have shown that individuals with calluses exhibit markedly elevated plantar pressures compared to the callus-free individuals (9, 10). The updated 2023 IWGDF guidelines emphasize the critical role of properly designed offloading footwear in preventing foot ulcers, particularly in diabetic patients who have a history of recurrent ulceration (11). However, Veves et al. reported that only 38% of DFU locations match the sites of peak plantar pressure (12). Scirè et al. found that despite wearing custom therapeutic offloading shoes, 41% of patients continued to develop recurrent callus (13). These findings suggest that while offloading footwear is beneficial, it may not be sufficient on its own to prevent the recurrence of diabetic foot calluses. This gap in effectiveness may be attributed to the intervention’s limited consideration of shear stress, which also plays a significant role in callus development on the plantar surface.

Under normal physiological conditions, the keratinization of the foot skin acts as a protective mechanism to prevent deep tissues from being damaged under mechanical stress (14, 15). However, in elderly individuals, due to repetitive friction during weight-bearing activities or ill-fitting shoes, there is a tendency for excessive thickening of the cornified layer, which exerts pressure on the nerves in the dermis, leading to pain (16). It has been reported that the occurrence rates of hyperkeratosis of the plantar skin in the elderly, aged 65 and above, are ranged from 20% to 65% (17, 18). If these cornified lesions left untreated, they can cause damage to deeper tissues and ultimately lead to ulceration (19, 20). Despite the clinical significance, the extant body of research has predominantly concentrated on assessing plantar pressure during gait, leaving a notable gap in our understanding of shear forces and their impact on the aging population.

Shear stress on the plantar surface emerges as a pivotal element in the genesis of foot corns, particularly within the cohorts of diabetic patients and the elderly. Yet, contemporary research efforts have largely been directed towards mitigating plantar pressure to prevent DFUs and other foot-related pathologies, often neglecting the scrutiny of plantar shear stress. As the scientific community increasingly acknowledges the predictive value of shear forces in the development and exacerbation of DFUs, there is a burgeoning need for sophisticated multi-axis stress sensors to address this knowledge gap. However, at this stage, precise, efficient, and reproducible measurement of plantar shear force distribution remains a substantial technical hurdle. This challenge is one of the key factors contributing to the relative neglect of plantar shear forces in past investigations.

Thorough research into the multidimensional aspects of foot biomechanics, including compression and shear, is paramount. It can help optimize the design of intervention measures, facilitate the creation of cushioning devices or insoles tailored for the elderly and individuals with diabetes, and redistribute loads from high-pressure areas, thereby alleviating foot discomfort, diminishing the incidence of calluses, and lowering the risk of ulcers. The hypothesis of the current study is that diabetic and healthy older (HO) individuals will exhibit significantly higher static and dynamic plantar pressures and shear forces compared to healthy younger (HY) adults. Consequently, the objectives of this study are as follows: (1) to compare the distributions vertical pressure and horizontal shear force during static standing among HY, HO and diabetic subjects; (2) to examine and compare the differences on biomechanical distribution in vertical pressure, anterior-posterior (AP) shear, and medial-lateral (ML) shear aspects during a complete gait cycle across the three groups of subjects.

## 2 Materials and methods

### 2.1 Subjects enrolling

This study included the following three groups of participants as the subjects: (1) HY group: individuals aged between 18 and 40 without a history of diabetes; (2) HO group: individuals aged between 50 and 70 without a history of diabetes; (3) diabetes group: individuals aged between 50 and 70 and diagnosed with type-II diabetes according to the diagnostic criteria outlined in the “Chinese Guidelines for the Prevention and Treatment of Type 2 Diabetes (2020 edition)” (21).

Inclusion criteria were as follows: (1) adults aged between 18 and 70 years; (2) ability to independently complete a normal gait cycle and cooperate with physical examinations and relevant tests; (3) absence of lower limb conditions other than diabetes that may affect plantar mechanical distribution; (4) absence of foot ulcers or history of foot ulcers in diabetic patients; (5) this study was designed as a cross-sectional study. Exclusion criteria were as follows: (1) presence of lower limb conditions other than diabetic foot, such as foot and ankle deformities (pes planus, cavus foot, hallux valgus, hammer toe, claw toe, and Charcot foot), plantar fasciitis, stroke, knee/ankle arthritis, or any other condition that may affect gait; (2) presence of foot ulcers.

**Figure 1:**
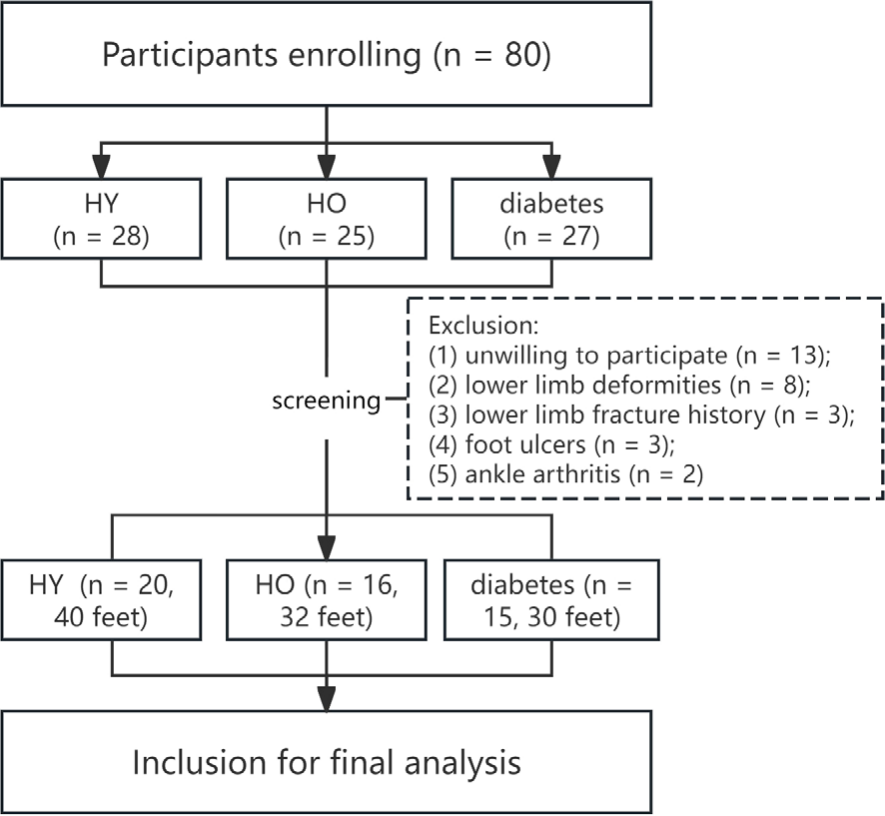
Flow chart of the participants enrollment. HY, healthy younger; HO, healthy older.

The participation of the subjects strictly adhered to the Helsinki Declaration and was approved by the Institutional Ethics Review Committee (approval number: KHLL2024-KY134). Written informed consent was mandatory from all individuals prior to their inclusion in the study. The flow diagram in **Figure 1** describes the participants’ enrollment process. The current study was conducted and reported according to the STROBE checklist (as shown in **Appendix 1**).

### 2.2 Measurement of the plantar 3D mechanics

A custom-built force platform, consisting of a pressure mat with 2400 sensing elements mounted flush onto a high-precision six-axis force/moment plate (600 × 400 mm^2^), was used for gathering the plantar mechanical data. The force plate has been employed in our previous studies (22, 23), and at the initial stage of designing the experiment, we conducted rigorous validation tests on the devices to confirm their accuracy. A six degrees of freedom disturbance platform, customized WIN06-010A standard model by Shanghai Yinghao Mechanical & Electrical Equipment Co., was used to adjust the degree of slope plane between force plate and ground. We also designed and fabricated two gait platforms of 1.5m*0.75m in size, which could embed the force plate in the central location. Before commencing data collection, a researcher provided comprehensive guidance to the participants, ensuring they fully understood the experimental procedures and could perform the necessary movements with proficiency, with the objective of capturing their natural gait as faithfully as possible. All data acquisition was performed at a sampling rate of 100 frames per second. Each process was repeated three times, and the average of the three repetitions was calculated.

**Figure 2:**
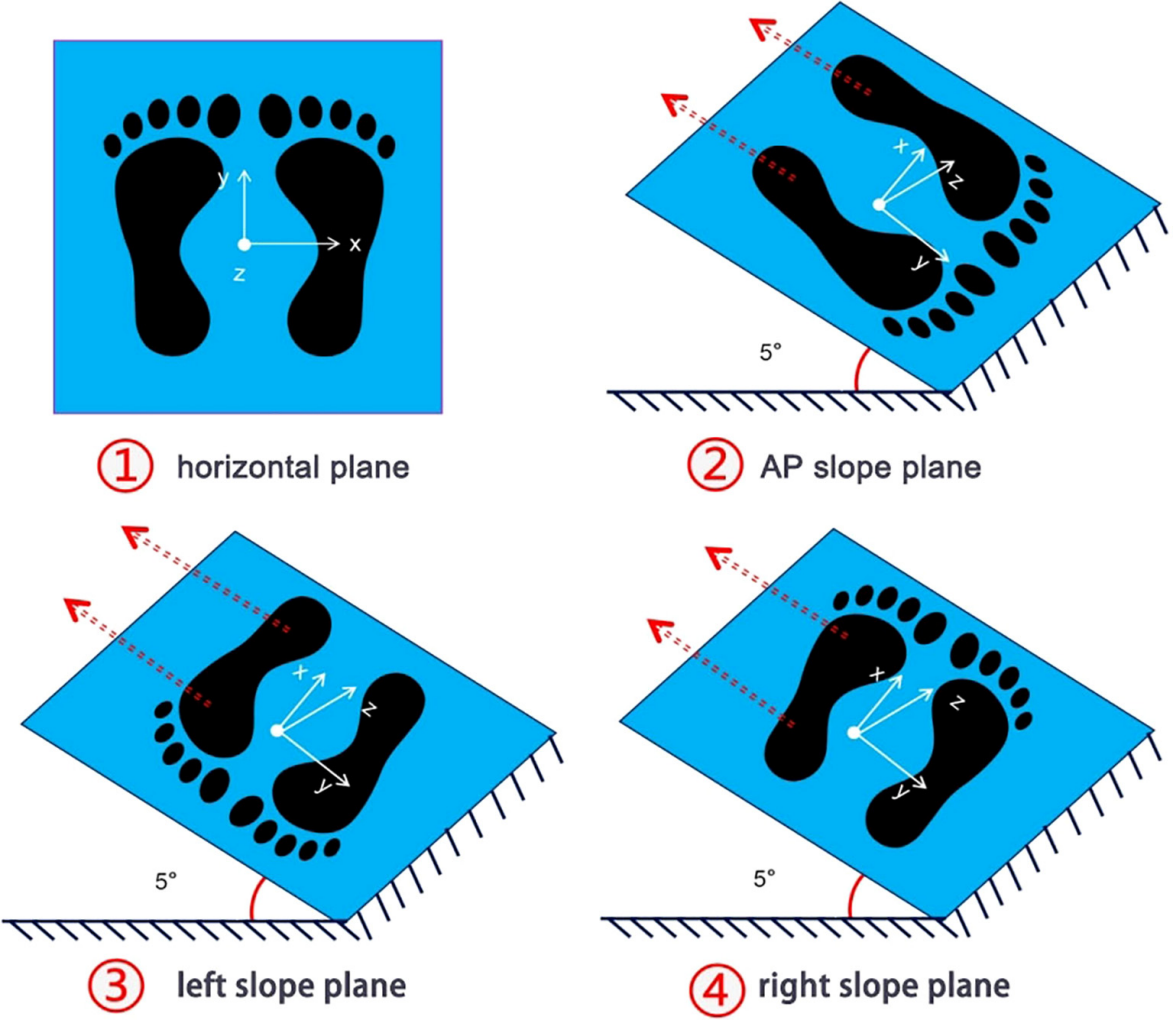
Illustrative diagram depicting the testing steps for the static 3D mechanical distribution of the plantar foot. ① Horizontal standing: Measurement of vertical pressure distribution on the plantar foot. ② Anterior-posterior (AP) slope standing: The force plate was adjusted to a 5° slope in the AP direction using a perturbation platform, and the distribution of static shear forces in different regions of the plantar foot is measured. ③-④ Left/right slope standing: The force plate was adjusted to a 5° slope on the left or right side using a perturbation platform, and the distribution of static shear forces in different areas of the plantar foot was tested. During each test, the subjects stood naturally in an upright position on the force plate, maintaining rest for 30 seconds, with the middle 15 seconds captured for analysis.

Participants exposed their lower legs and ankles and stand statically on the force plate, and finished the following four steps to test the static plantar mechanical distribution (see **Figure 2**): (1) horizontal plane: the force plate was placed horizontally on the ground, and the static normal pressure was collected; (2) slope planes: an AP/left/right angle of 5° between force plate and horizontal plane was created, to collect the AP/ML (left foot)/ML (right foot) static shear force distributions. The angle between force plate and horizontal plane was adjusted by six-degree of freedom disturbance platform. Participants stood statically on the force plate in a natural posture, maintaining a resting position for 30 seconds.

As shown in **Supplementary Figure 1**, to observe the dynamic plantar mechanical distribution, the participants walked along the gait platform (3m*0.75m, composed of two sections of 1.5m*0.75m platform) at a steady pace, using their habitual walking frequency and stride length. The walking pattern utilized the “two-step method”, and on the second step the heel to be tested would make contact with the central part of force plate embedded horizontally within the gait platform. The collection of three-dimensional dynamic plantar mechanical data for a single foot was completed during one gait cycle. Then, the same step was followed to collect data for the other feet.

### 2.3 Data processing

Utilizing a custom MATLAB program, the footprint was generated by superimposing cloud images of different frames during the measurement period. Then, principal component analysis (PCA) was applied to identify the primary axis direction and central coordinates of the footprint. Following this, based on predefined ratios, the footprint were automatically partitioned into seven different rectangular regions (**Supplementary Figure 2**): the hallux, 2^nd^-5^th^ toes (T_2-5_), 1^st^ metatarsal head (M_1_), 2^nd^-3^rd^ metatarsal heads (M_2-3_), 4^th^-5^th^ metatarsal heads (M_4-5_), lateral arch region (LA), and heel regions. The data processing was similar to our previous study (22). Static plantar pressure and shear force data were collected for 30 seconds, with the central 15 seconds of stable stance selected for averaging purposes. During the gait cycle, peak pressures and peak shear forces in the AP and ML directions were determined. These peak values correspond to the highest points along the time-force curve. The areas under the time-force curve were calculated in three dimensions, yielding indices for vertical force-time integration (FTI), AP shear FTI, and ML shear FTI. To eliminate the influence of body weight on plantar mechanical distribution, the aforementioned indices were all normalized using body weight:


(1) $$F_{S}=\frac{F_{0}}{0.01*W}$$

(F_0_ is the plantar mechanical index before standardization; W is the body weight of the subject; Fs is the plantar mechanical index obtained after standardization).

### 2.4 Statistical analysis

Assuming an alpha level of 0.05 based on preliminary pilot study, the required sample size per group was calculated to achieve a power of 0.80 for detecting statistically significant differences in the distributions of vertical pressure and shear force across the three groups. This analysis indicated a minimum requirement of n = 30 (30 feet, 15 participants) per group to meet the desired power criterion.

We employed “mean ± standard deviation (MD)” to characterize the mechanical parameters across various plantar regions. Categorical baseline data was compared among the three groups, using either Pearson’s chi-square test or Fisher’s exact test, contingent upon theoretical frequency table. Regarding continuous baseline data and comparisons of mechanical distribution among different plantar regions in the three groups, the following approaches were applied: (1) for data exhibiting a normal distribution and meeting the homogeneity of variance (HoV) assumption, one-way analysis of variance (ANOVA) was used, followed by the SNK-q test for *post hoc* pairwise comparisons; (2) if the data followed a normal distribution but do not satisfy the assumption of HoV, the Brown-Forsythe (BF) test was used, followed by the Tamhane’s T_2_ test for *post hoc* pairwise comparisons; (3) if the data did not follow a normal distribution, the non-parametric Kruskal-Wallis H test was used, followed by the Dunnett’s test for *post hoc* pairwise comparisons.

All data analyses were performed using R language version 4.2.2 (Foundation for Statistical Computing, Vienna, Austria). A significance level of p<0.05 (two-tailed) was considered statistically significant. To evaluate the normality and the HoV assumptions, we used the Shapiro-Wilk test (W-test) and the modified Bartlett’s test, considering p<0.05 as indicative of non-normality and a breach of the HoV assumption.

## 3 Results

### 3.1 Baseline characteristics of the enrolled participants

The demographic information of the three groups is shown in **Table 1**. Twenty (40 feet), 16 (32 feet) and 15 (30 feet) subjects were enrolled in HY, HO and patients with diabetes groups respectively. No substantial differences were observed in gender distribution across the groups. However, a statistically significant elevation in mean age was noted in both the HO and patients with diabetes groups compared to the HY group. Age parity was established between the HO and patients with diabetes groups. A significantly higher BMI was found in patients with diabetes group than HY adults, and no significant difference in incidence of plantar callus was found among the three groups.

**Table 1:** Comparison results of demographic of the three groups of subjects.

Demographic	Group			Comparison among three groups	A vs. B	A vs. C	B vs. C
	Healthy younger group (A) (20cases/40 foot)	Healthy older group (B) (16 cases/32 foot)	Patients with diabetes group (C) (15 cases/30 foot)				
**Sex**							
male	11 (55.0%)	7 (43.75%)	7 (46.67%)	X^2^ = 0.497; P = 0.780	X^2^ = 0.450; P = 0.502	X^2^ = 0.238; P = 0.625	X^2^ = 0.027; P = 0.870
female	9 (45.0%)	9 (56.25%)	8 (53.33%)
**Age** (year)	27.65±5.60	58.88±3.54	58.33±5.33	F = 236.765; P<0.001***	MD=-31.23; P<0.001***	MD=-30.68; P<0.001***	MD=0.54; P=0.754
**BMI** (kg/m^2^)	22.01±2.18	23.31±2.35	24.88±4.43	H = 7.939; P = 0.019*	D = 1.607; P = 0.186	D = 2.741; P = 0.012*	D = 0.689; P = 0.710
**Smoking**							
Yes	6 (30.00%)	6 (37.50%)	5 (33.33%)	X^2^ = 0.225; P = 0.894	X^2^ = 0.226; P = 0.635	X^2^ = 0.044; P = 0.833	X^2^ = 0.059; P = 0.809
No	14 (70.00%)	10 (62.50%)	10 (66.67%)
**Drinking**							
Yes	6 (30.00%)	5 (31.25%)	3 (20.00%)	Fisher’s; P = 0.789	Fisher’s; P = 1.000	Fisher’s; P = 0.700	Fisher’s; P = 0.685
No	14 (70.00%)	11 (68.75%)	12 (80.00%)
**Hypertension**							
Yes	2 (10.00%)	8 (50.00%)	10 (66.67%)	X^2^ = 12.684; P = 0.002**	Fisher’s; P = 0.011*	X^2^ = 12.216; P<0.001***	X^2^ = 0.883; P = 0.347
No	18 (90.00%)	8 (50.00%)	5 (33.33%)
**Coronary heart disease**							
Yes	0 (0.00%)	0 (0.00%)	1 (6.67%)	Fisher’s; P = 0.294	NA	Fisher’s; P = 0.429	Fisher’s; P = 0.484
No	20 (100.00%)	16 (100.00%)	14 (93.33%)
**Plantar callus**							
Yes	8 (40.00%)	4 (25.00%)	8 (53.33%)	X^2^ = 2.616; P = 0.270	X^2^ = 0.900; P = 0.343	X^2^ = 0.614; P = 0.433	X^2^ = 2.620; P = 0.106
No	12 (60.00%)	12 (75.00%)	7 (46.67%)
Average	2.13±0.99	2.25±1.26	2.63±0.92	H = 2.217; P = 0.330	D = 0.250; P = 0.958	D = 1.511; P = 0.234	D = 0.622; P = 0.692
**Heel callus**							
Yes	2 (10.00%)	0 (0.00%)	1 (6.67%)	Fisher’s; P = 0.624	Fisher’s; P = 0.492	Fisher’s; P = 1.000	Fisher’s; P = 0.484
No	18 (90.00%)	16 (100.00%)	14 (93.33%)

X^2^, Pearson's Chi square test; F, univariate Analysis of Variance; H, Kruskal-Wallis H test; D, the effect size of Dunnett's test;MD(mean difference), the mean of the difference between two groups of continuous variables; Fisher's exact probability method is used as an alternative when Pearson's Chi-square test is not satisfied. *P<0.05, **P<0.01, ***P<0.001.

### 3.2 Correlation between diabetes and aging and the plantar static mechanical distribution

**Figure 3:**
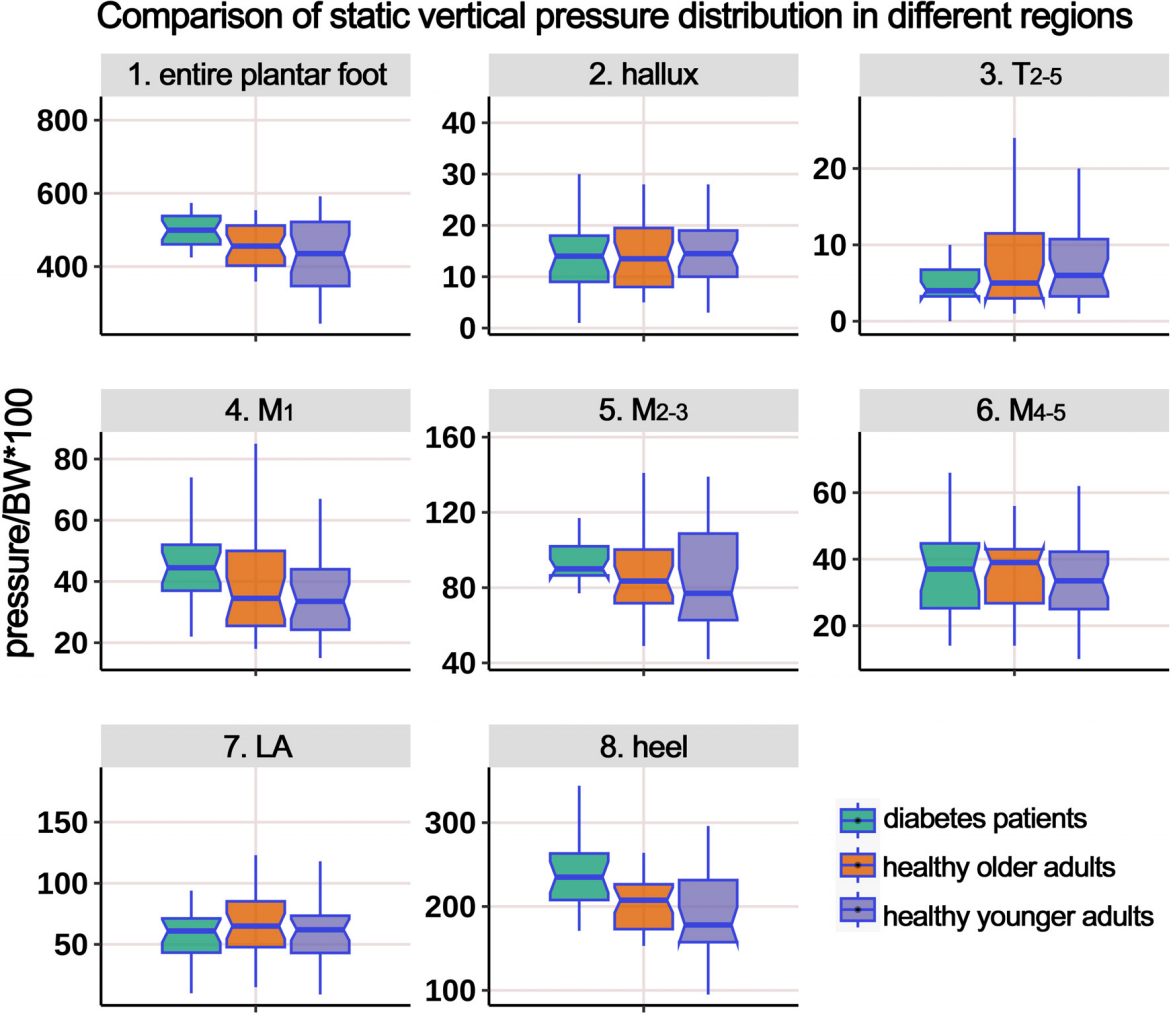
Comparison of static vertical pressure distribution in different regions of the plantar foot among healthy younger, healthy older, and patients with diabetes groups. The data from all three groups were standardized by body weight (BW) to eliminate the influence of varying body weights on pressure distribution. T_2-5_, 2^nd^-5^th^ toes; M_1_, 1^st^ metatarsal head; M_2-3_, 2^nd^-3^rd^ metatarsal heads; M_4-5_, 4^th^-5^th^ metatarsal heads; LA, lateral arch region.

**Table 2** shows the static pressure distribution in the entire plantar foot and seven regional areas. The mechanical data in all regions was standardized based on body weight, and a boxplot (**Figure 3**) was created. The ANOVA revealed significant differences in the standardized static pressure among the three groups for the entire plantar foot, M_1_, M_2-3_, and heel regions. *Post hoc* pairwise comparisons showed that compared to HY individuals, the standardized static pressure significantly increased in the M_2-3_ and heel regions in the HO group. Similarly, compared to HY individuals, diabetic patients exhibited significantly higher standardized static pressure in the entire plantar foot, M_1_, M_2-3_, and heel regions.

**Table 2:** Comparison of the static vertical pressure distribution of different plantar regions.

Regions	Group A (N)	Group B (N)	Group C (N)	P value (overall)	P value (A vs. B)	P value (A vs. C)	P value (B vs. C)
entire plantar	301.99±56.98	284.72±54.35	336.60±52.37	0.049^F*^	0.073	0.041*	0.514
hallux	10.59±4.20	9.31±4.31	10.07±5.73	0.991^H^	0.995	0.989	0.993
T_2-5_	4.97±3.28	5.30±3.79	3.90±2.82	0.279^H^	0.293	0.226	0.982
M_1_	26.52±8.01	23.79±9.72	30.86±8.81	0.035^H*^	0.069	0.027*	0.954
M_2-3_	60.93±15.08	54.41±14.61	65.66±15.04	0.022^H*^	0.026*	0.044*	0.708
M_4-5_	22.11±8.22	23.49±8.88	24.58±8.84	0.854^F^	0.820	0.765	0.858
LA	44.53±26.73	42.68±19.30	40.41±20.65	0.689^H^	0.622	0.890	0.861
heel	132.35±27.53	126.01±27.45	161.10±33.07	0.002^BF**^	0.003**	0.001**	0.563

Group A: healthy younger subjects; group B: healthy older subjects; group C: diabetic subjects. F, H and BF represent the effect sizes of one-way ANOVA, Kruskal-Wallis H test, and Brown-Forsythe test, respectively. SNK-q, Dunnett’s and Tamhane’s T_2_ tests were used for *post-hoc* multiple comparisons corresponding to the three statistical analyses. The data are presented as “mean±SD”. T_2-5_: 2^nd^-5^th^ toes; M_1_, 1^st^ metatarsal head; M_2-3_, 2^nd^-3^rd^ metatarsal heads; M_4-5_, 4^th^-5^th^ metatarsal heads; LA, lateral arch region. *P<0.05, **P<0.01.

**Figure 4:**
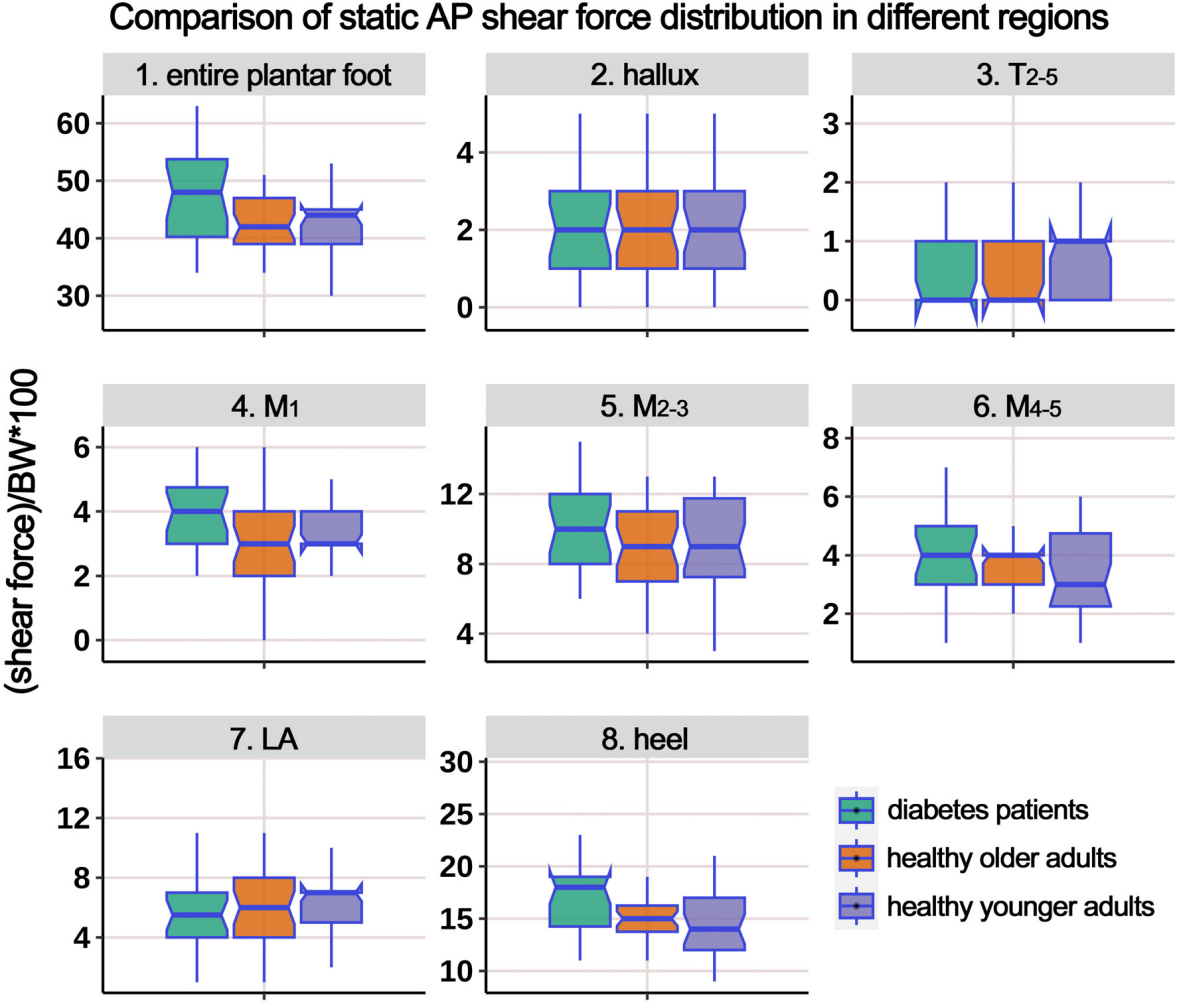
Comparison of static anterior-posterior (AP) shear force distribution in different regions of the plantar foot among healthy younger, healthy older, and patients with diabetes groups. The data from all three groups were standardized by body weight (BW) to eliminate the influence of varying body weights on shear force distribution. T_2-5_, 2^nd^-5^th^ toes; M_1_, 1^st^ metatarsal head; M_2-3_, 2^nd^-3^rd^ metatarsal heads; M_4-5_, 4^th^-5^th^ metatarsal heads; LA, lateral arch region.

**Table 3** presents the distribution of AP static shear force in the entire plantar foot and the seven regional areas. Similar to the previous analysis, the data were standardized based on body weight, and a boxplot (**Figure 4**) was generated. The ANOVA results indicated significant differences in the standardized static AP shear force among the three groups for the entire plantar foot, T_2-5_, M_1_, and heel regions. *Post hoc* pairwise comparisons showed that compared to HY individuals, the standardized AP shear force significantly increased in the entire plantar foot and M_1_ region in the HO group. Similarly, compared to HY individuals, diabetic patients exhibited significantly higher standardized AP shear force in the entire plantar foot, T_2-5_, M_1_, M_2-3_, and heel regions.

**Table 3:** Comparison of the static anterior-posterior shear force distribution of different plantar regions.

Regions	Group A (N)	Group B (N)	Group C (N)	P value (overall)	P value (A vs. B)	P value (A vs. C)	P value (B vs. C)
entire plantar	28.44±5.65	26.94±5.19	31.99±5.97	0.022^F*^	0.015*	0.024*	0.851
hallux	1.68±0.74	1.59±0.70	1.72±0.86	0.997^H^	0.995	1.000	0.998
T_2-5_	0.80±0.44	0.72±0.45	0.53±0.37	0.034^H*^	0.083	0.024*	0.833
M_1_	2.38±0.80	2.24±0.78	2.99±0.85	0.033^F*^	0.039*	0.019*	0.922
M_2-3_	6.37±1.77	5.66±1.58	7.06±1.84	0.068^F^	0.079	0.037*	0.943
M_4-5_	2.64±1.11	2.59±0.88	2.92±1.02	0.590^F^	0.647	0.546	0.555
LA	4.72±2.51	4.13±1.83	4.49±2.15	0.874^F^	0.859	0.761	0.879
heel	9.87±2.35	10.02±2.59	12.28±3.42	0.024^H*^	0.137	0.030*	0.434

Group A: healthy younger subjects; group B: healthy older subjects; group C: diabetic subjects. F and H represent the effect sizes of one-way ANOVA and Kruskal-Wallis H test, respectively. SNK-q test and Dunnett’s test were used for *post-hoc* multiple comparisons corresponding to the two statistical analyses. The data are presented as “mean±SD”. T_2-5_: 2^nd^-5^th^ toes; M_1_, 1^st^ metatarsal head; M_2-3_, 2^nd^-3^rd^ metatarsal heads; M_4-5_, 4^th^-5^th^ metatarsal heads; LA, lateral arch region. *P<0.05.

**Figure 5:**
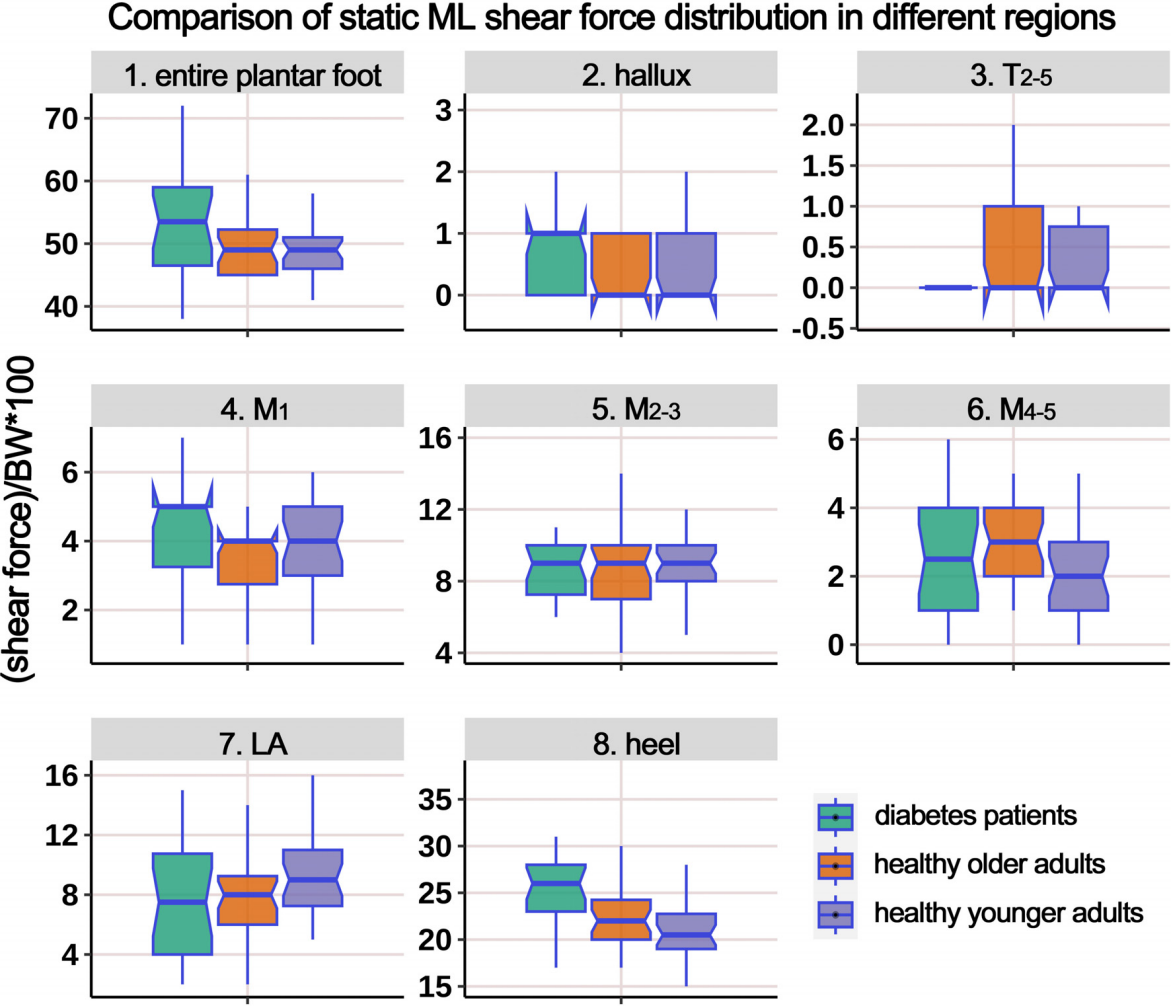
Comparison of static medial-lateral (ML) shear force distribution in different regions of the plantar foot among healthy younger, healthy older, and patients with diabetes groups. The data from all three groups were standardized by body weight (BW) to eliminate the influence of varying body weights on shear force distribution. T_2-5_, 2^nd^-5^th^ toes; M_1_, 1^st^ metatarsal head; M_2-3_, 2^nd^-3^rd^ metatarsal heads; M_4-5_, 4^th^-5^th^ metatarsal heads; LA, lateral arch region.

**Table 4** displays the distribution of ML static shear force in the entire plantar foot and the seven regional areas. After standardizing the data based on body weight, a boxplot (**Figure 5**) was constructed. The results revealed significant differences in the standardized static ML shear force among the three groups for the entire plantar foot, M_4-5_, and heel regions. *Post hoc* pairwise comparisons showed that compared to HY individuals, the standardized ML shear force significantly increased in the entire plantar foot, T_2-5_, and M_1_ regions in the HO group. Similarly, compared to HY individuals, diabetic patients exhibited significantly higher standardized ML shear force in the M_4-5_ region.

**Table 4:** Comparison of the static medial-lateral shear force distribution of different plantar regions.

Regions	Group A (N)	Group B (N)	Group C (N)	P value (overall)	P value (A vs. B)	P value (A vs. C)	P value (B vs. C)
entire plantar	32.68±6.45	30.94±4.10	36.27±7.53	0.031^BF*^	0.019*	0.014*	0.620
hallux	0.65±0.49	0.66±0.36	0.80±0.49	0.465^H^	0.728	0.506	0.636
T_2-5_	0.52±0.32	0.52±0.29	0.38±0.23	0.058^H^	0.038*	0.127	0.969
M_1_	2.94±0.69	2.50±0.85	3.39±1.09	0.055^F^	0.039*	0.207	0.226
M_2-3_	6.23±1.48	5.60±1.22	6.54±1.85	0.637^H^	0.640	1.000	0.658
M_4-5_	1.65±1.02	2.08±0.77	2.02±1.05	0.008^F**^	0.962	0.009**	0.022*
LA	6.57±2.55	5.17±1.96	5.63±2.95	0.146^F^	0.991	0.221	0.100
heel	14.11±3.00	14.37±2.67	17.50±4.14	<0.001^H***^	0.074	0.002**	0.117

Group A: healthy younger subjects; group B: healthy older subjects; group C: patients with diabetes. F, H and BF represent the effect sizes of one-way ANOVA, Kruskal-Wallis H test, and Brown-Forsythe test, respectively. SNK-q, Dunnett's and Tamhane’s T_2_ tests were used for *post-hoc* multiple comparisons corresponding to the three statistical analyses. The data are presented as “mean±SD”. T_2-5_: 2^nd^-5^th^ toes; M_1_, 1^st^ metatarsal head; M_2-3_, 2^nd^-3^rd^ metatarsal heads; M_4-5_, 4^th^-5^th^ metatarsal heads; LA, lateral arch region.*P<0.05,**P<0.01, ***P<0.001.

### 3.3 Correlation between diabetes and aging and the plantar mechanical distribution during gait

#### 3.3.1 Pressure-time integral (PTI) and peak pressure

**Figure 6:**
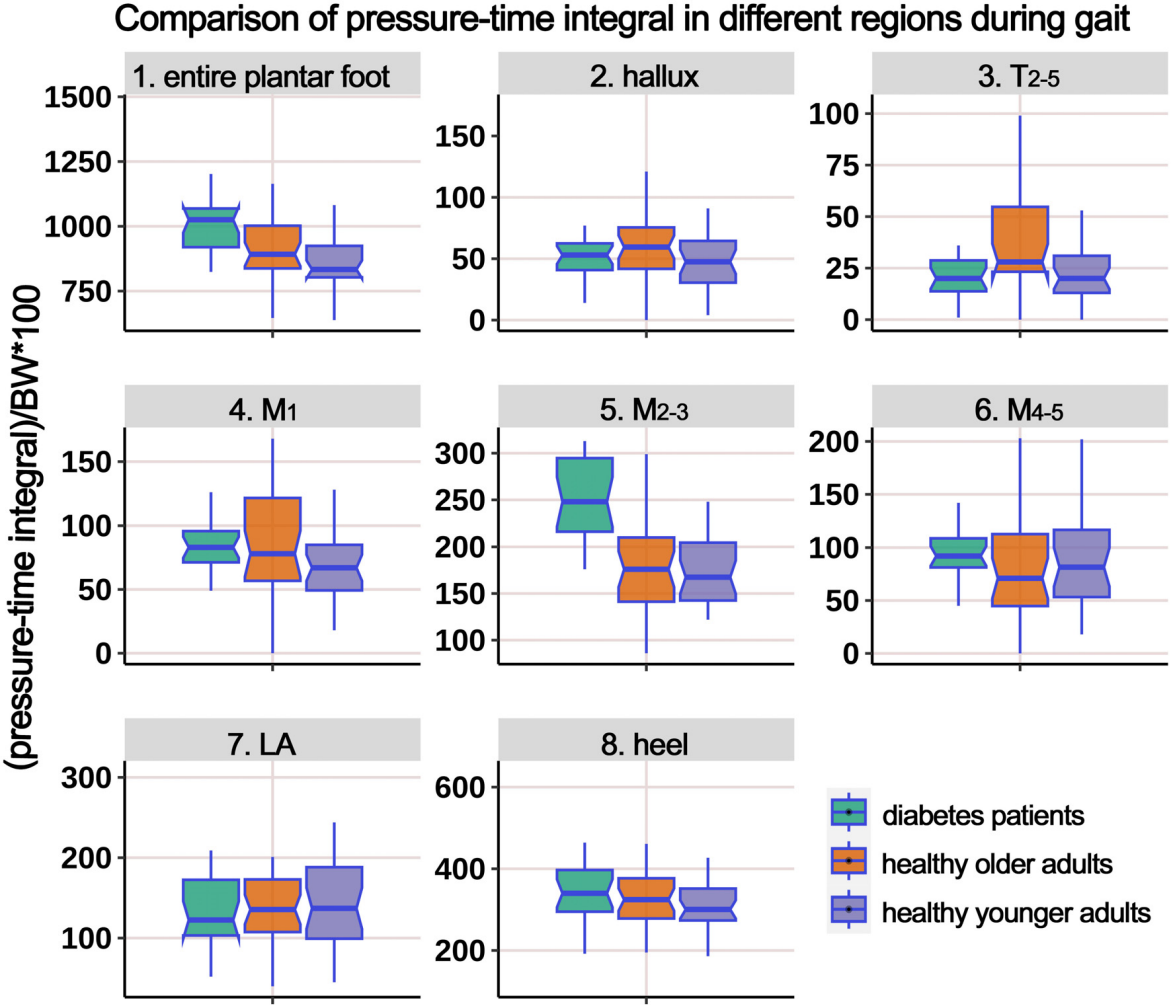
Comparison of pressure-time integral in different plantar regions during gait cycle among healthy younger adults, healthy older adults, and diabetes patients. The data from all three groups were normalized by body weight (BW) to minimize the impact of weight differences. T_2-5_, 2^nd^-5^th^ toes; M_1_, 1^st^ metatarsal head; M_2-3_, 2^nd^-3^rd^ metatarsal heads; M_4-5_, 4^th^-5^th^ metatarsal heads; LA, lateral arch region.

**Supplementary Table 1** presents the distribution of PTI in different plantar regions during the gait cycle, and **Figure 6** illustrates the boxplot following body weight calibration. The results showed significant differences among the groups in the entire plantar, T_2-5_, and M_2-3_ regions. *Post-hoc* multiple comparisons revealed the following: (i) Compared to HY participants, HO individuals had significantly higher PTI in the entire plantar, T_2-5_, M_2-3_, and M_4-5_ regions; (ii) compared to HY participants, individuals with diabetes had significantly elevated PTI in the entire plantar, M_1_, and M_2-3_ regions; (iii) compared to HO individuals, individuals with diabetes showed a significant decrease in PTI in the region of T_2-5_.

**Figure 7:**
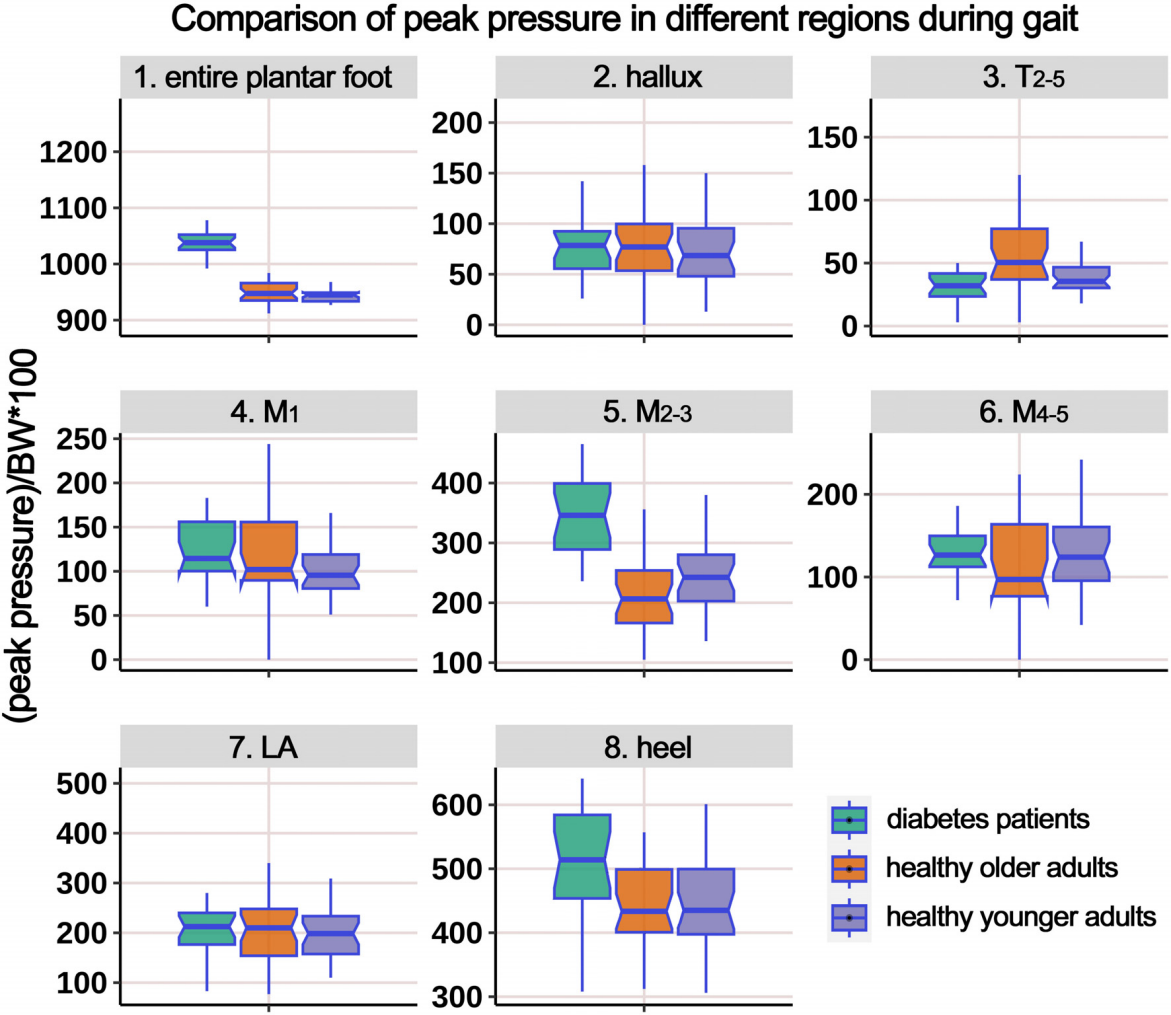
Comparison of peak pressure in different plantar regions during gait cycle among healthy younger adults, healthy older adults, and diabetes patients. The data from all three groups were normalized by body weight (BW) to minimize the impact of weight differences. T_2-5_, 2^nd^-5^th^ toes; M_1_, 1^st^ metatarsal head; M_2-3_, 2^nd^-3^rd^ metatarsal heads; M_4-5_, 4^th^-5^th^ metatarsal heads; LA, lateral arch region.

**Supplementary Table 2** presents the distribution of PP during the gait cycle, and **Figure 7** illustrates the boxplot after body weight standardization. The results showed significant differences among the groups in the entire plantar, T_2-5_, M_1_, M_2-3_, M4-5, and heel regions. *Post-hoc* multiple comparisons revealed the following: (i) compared to HY participants, HO individuals had significantly higher PP in the entire plantar, M_1_, and heel regions. However, the PP below the M_2-3_ region significantly decreased; (ii) compared to HY participants, individuals with diabetes demonstrated significantly increased PP in the entire plantar, T_2-5_, M_2-3_, M_4-5_, and heel regions; (iii) compared to HO individuals, diabetes group exhibited a significant decrease in PP in the T_2-5_ region, while the PP below the M_2-3_ region significantly increased.

#### 3.3.2 AP shear FTI and peak shear force (PSF)

**Figure 8:**
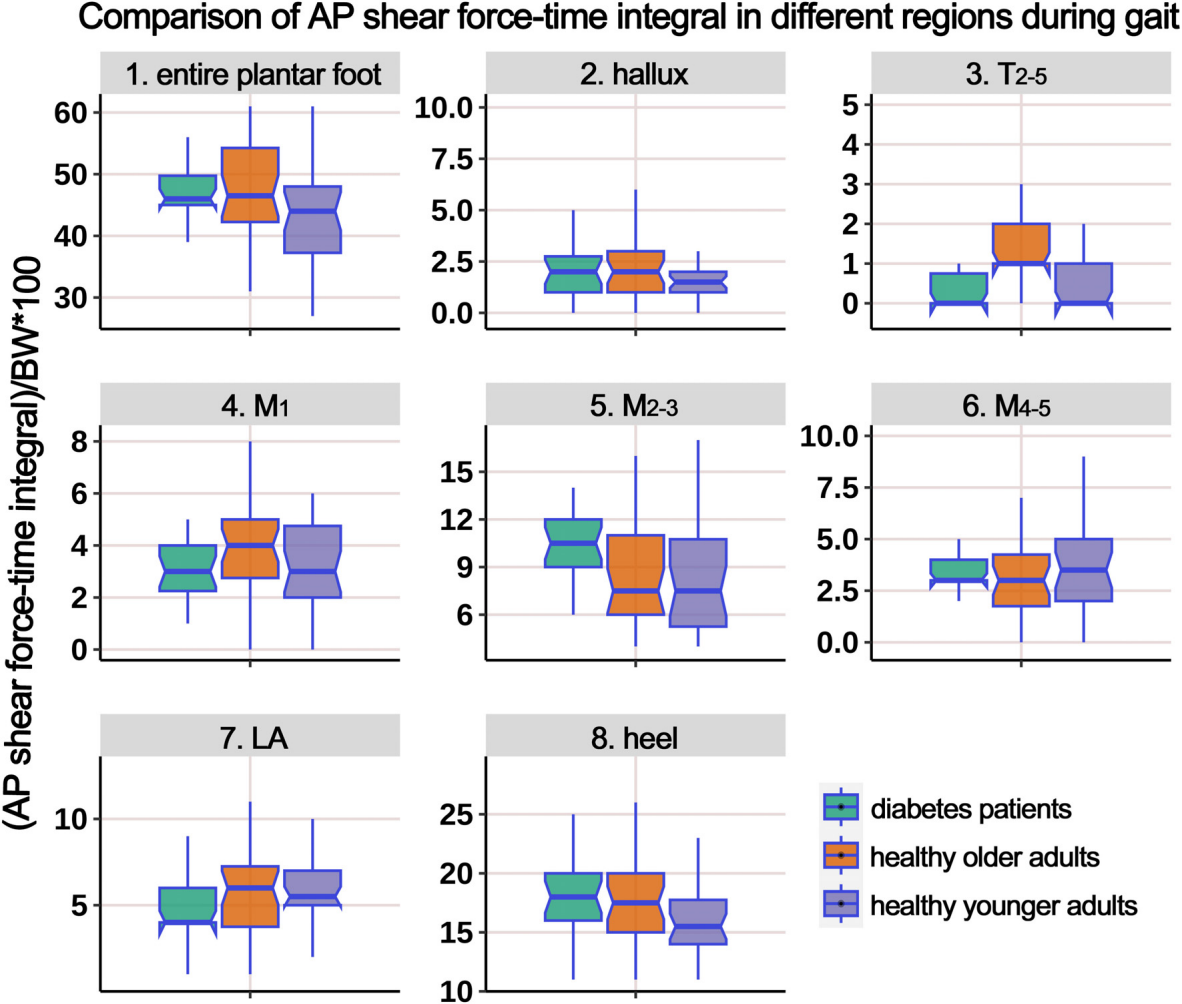
Comparison of anterior-posterior shear force-time integral in different plantar regions during gait cycle among healthy younger adults, healthy older adults, and diabetes patients. The data from all three groups were normalized by body weight (BW) to minimize the impact of weight differences. T_2-5_, 2^nd^-5^th^ toes; M_1_, 1^st^ metatarsal head; M_2-3_, 2^nd^-3^rd^ metatarsal heads; M_4-5_, 4^th^-5^th^ metatarsal heads; LA, lateral arch region.

**Supplementary Table 3** presents the distribution of AP shear FTI at different plantar regions during gait, and **Figure 8** illustrates the boxplot after body weight standardization. The comparison showed significant differences among the groups in the entire plantar, T_2-5_, M_1_, M_2-3_, and heel region. *Post-hoc* multiple comparisons revealed the following: (i) compared to HY participants, HO individuals showed a significant increase in shear FTI in the regions of T_2-5_, M_1_, and M_2-3_; (ii) compared to HY participants, individuals with diabetes exhibited significantly higher shear FTI in the entire plantar, M_2-3_, and heel regions; (iii) compared to HO individuals, diabetes group had significant lower shear FTI in the T_2-5_ and M_1_ regions (P = 0.016*), while the entire plantar shear FTI significantly increased.

**Figure 9:**
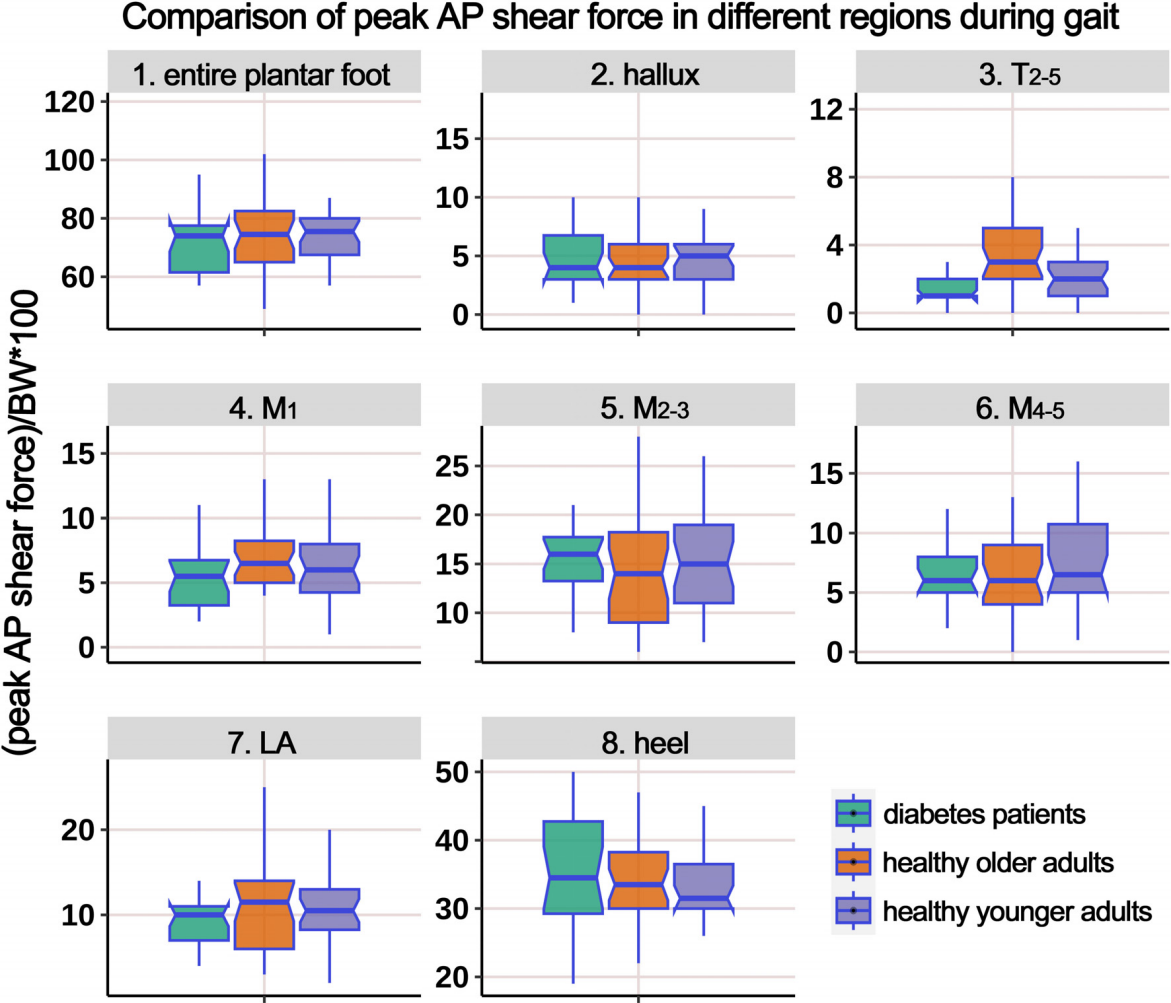
Comparison of peak anterior-posterior shear force in different plantar regions during gait cycle among healthy younger adults, healthy older adults, and diabetes patients. The data from all three groups were normalized by body weight (BW) to minimize the impact of weight differences. T_2-5_, 2^nd^-5^th^ toes; M_1_, 1^st^ metatarsal head; M_2-3_, 2^nd^-3^rd^ metatarsal heads; M_4-5_, 4^th^-5^th^ metatarsal heads; LA, lateral arch region.

**Supplementary Table 4** presents the distribution of AP PSF in different plantar regions during the gait cycle, and **Figure 9** illustrates the boxplot after body weight standardization. The results showed a significant difference among the groups only in the region of T_2-5_. *Post-hoc* multiple comparisons revealed that the HO individuals had significantly higher PSF in the T_2-5_ region compared to HY participants.

#### 3.3.3 ML shear FTI and PSF

**Figure 10:**
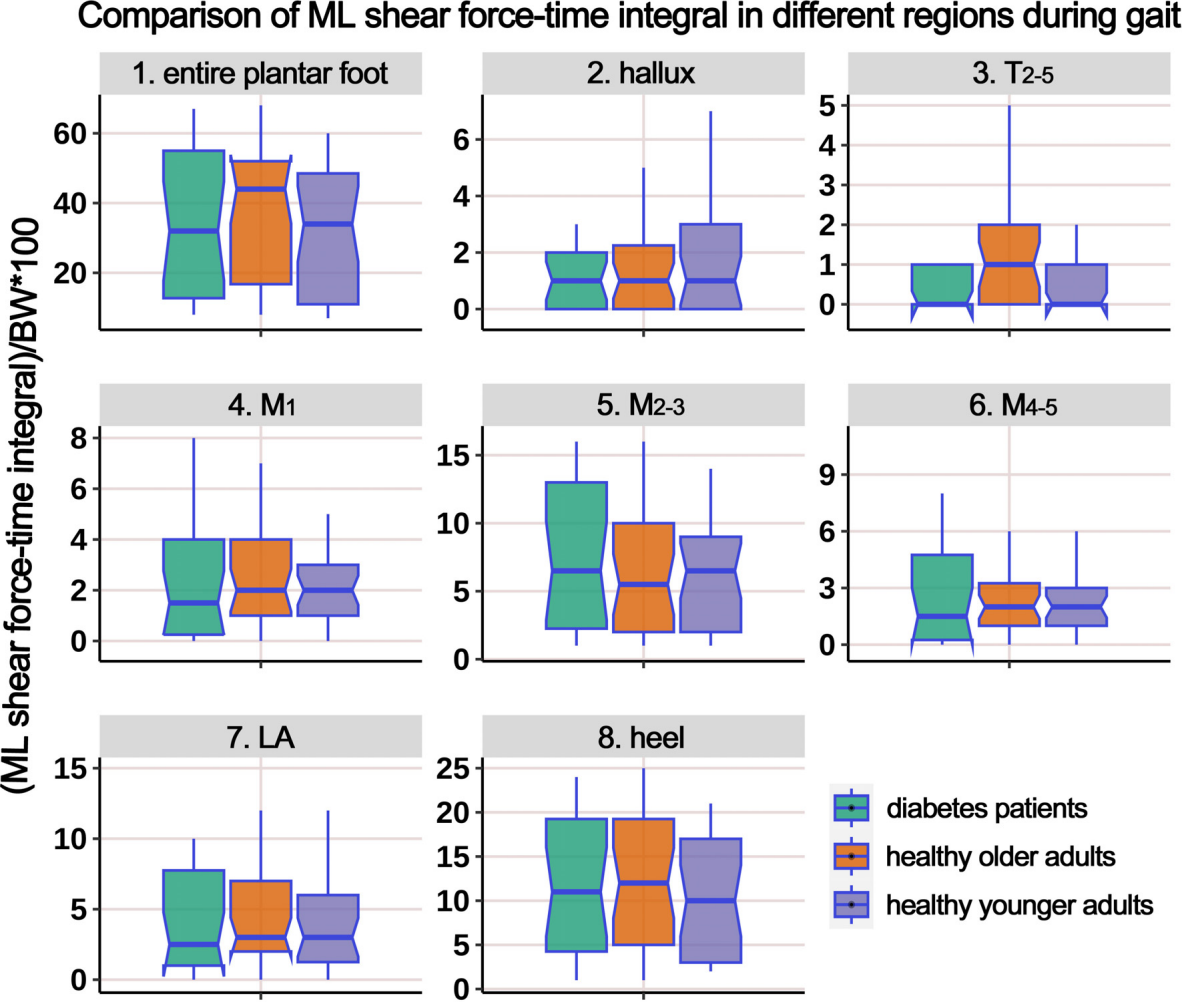
Comparison of medial-lateral shear force-time integral in different plantar regions during gait cycle among healthy younger adults, healthy older adults, and diabetes patients. The data from all three groups were normalized by body weight (BW) to minimize the impact of weight differences. T_2-5_, 2^nd^-5^th^ toes; M_1_, 1^st^ metatarsal head; M_2-3_, 2^nd^-3^rd^ metatarsal heads; M_4-5_, 4^th^-5^th^ metatarsal heads; LA, lateral arch region.

**Supplementary Table 5** presents the distribution of ML shear FTI in different plantar regions during the gait cycle, and **Figure 10** illustrates the boxplot after body weight standardization. The results showed a significant difference among the groups only in the T_2-5_ region. *Post-hoc* multiple comparisons revealed that the HO individuals had significantly higher ML shear FTI in the region of T_2-5_ compared to HY participants.

**Figure 11:**
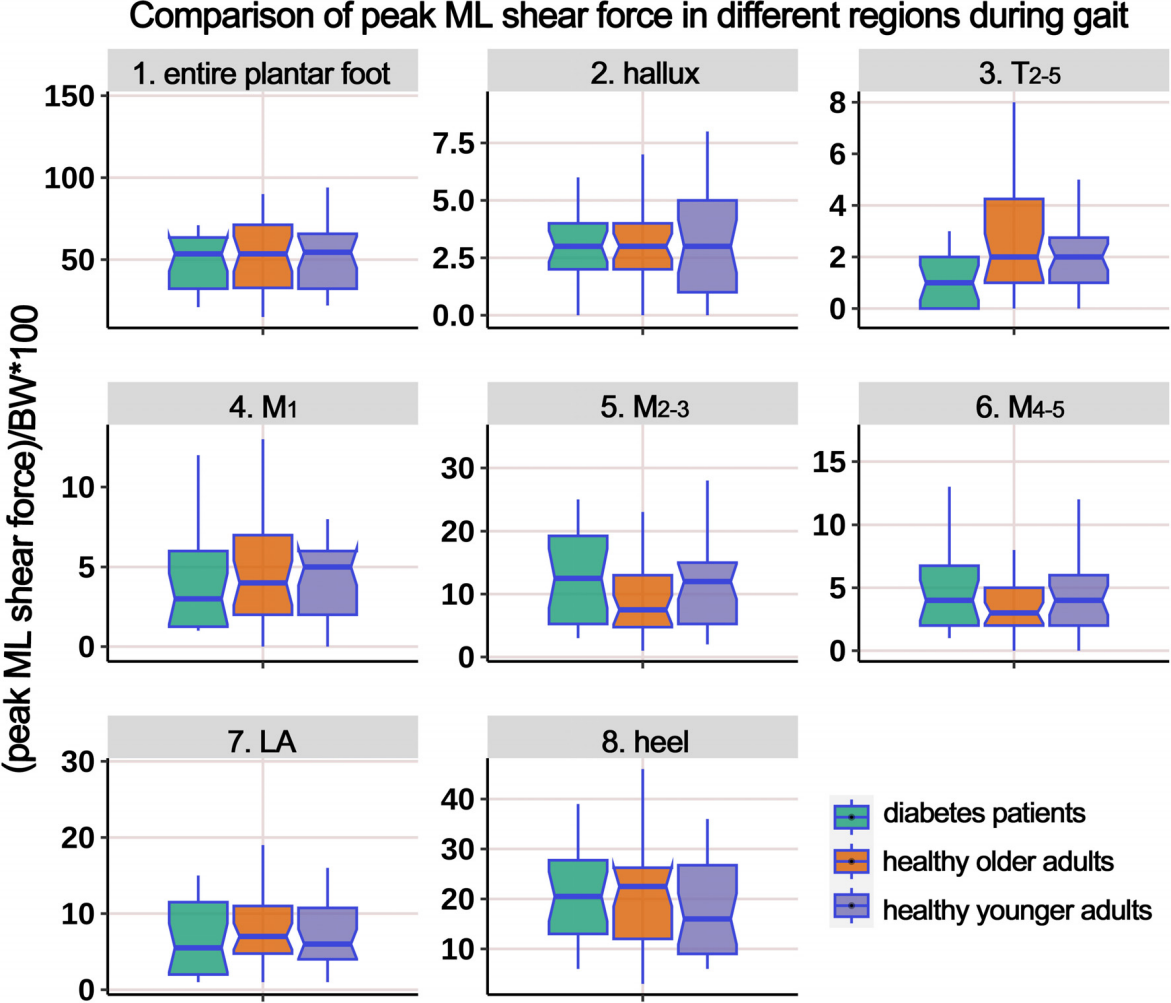
Comparison of peak medial-lateral shear force in different plantar regions during gait cycle among healthy younger adults, healthy older adults, and diabetes patients. The data from all three groups were normalized by body weight (BW) to minimize the impact of weight differences. T_2-5_, 2^nd^-5^th^ toes; M_1_, 1^st^ metatarsal head; M_2-3_, 2^nd^-3^rd^ metatarsal heads; M_4-5_, 4^th^-5^th^ metatarsal heads; LA, lateral arch region.

**Supplementary Table 6** presents the distribution of ML PSF in different regions of the plantar foot during the gait cycle. The boxplot in **Figure 11** represents the data after body weight standardization. The results indicate that, overall, there were no statistically significant differences in the ML PSF among the three groups. However, *post hoc* multiple comparisons revealed a significant increase in PSF in the T_2-5_ region in the HO group compared to the HY participants.

## 4 Discussion

In this investigation, we assessed and contrasted the three-dimensional plantar foot mechanical distribution in HY and HO adults, alongside diabetic patients, during static standing and gait cycle. The main findings include: (1) compared to HY individuals, both diabetes patients and HO adults exhibited increased static and dynamic pressures/shear forces; (2) upon subdividing the plantar foot into different regions, the most pronounced increases in stress were detected in the heel and the M_2-3_ regions, which highlights the need to focus on these regions to mitigate the risk of developing DFUs and alleviate foot discomfort in the geriatric population; (3) there was a significant increase in the time integral of pressure and AP shear forces during the gait cycle.

Currently, the systems used for plantar force testing can be classified into two main categories: plantar force plates and wearable force measurement devices (24, 25). Plantar force plates are generally used in gait laboratories and suitable for measuring quasi-static or limited dynamic (1-2 gait cycles) conditions, which cannot fully reflect the load on the plantar foot during daily activities. Instead, wearable devices are attached to the skin of the plantar foot using specific methods to enable continuous monitoring over multiple gait cycles. Wang et al. (26) reviewed wearable sensor systems for monitoring plantar foot loads in diabetic individuals and found that commercially available sensors can only measure plantar pressure. Sensors with shear force testing capabilities are still in the experimental stage. Future research trends will focus on the development of stable and reliable wearable multi-axial stress monitoring systems. Additionally, it is important to establish standardized methods for validating the accuracy and repeatability of sensors. Due to limitations in the development of wearable multi-dimensional force sensor technology, the repeatability of measurements using wearable sensors, and their commercial availability, this study employed a plantar force plate for three-dimensional stress testing.

In a study by Mueller et al. (27), a comparative analysis was undertaken between 12 subjects with diabetic peripheral neuropathy (DPN) and 12 non-diabetic controls. The analysis focused on the PP, PP gradient, peak shear stress, and peak shear stress depth. The findings revealed that all forefoot indexes were substantially elevated in the diabetic cohort (34% to 85% higher) compared to the normal subjects. In the study by Bacarin et al. (28), the impact of DFU history on plantar pressure distribution during the gait cycle was compared among 20 healthy subjects, 17 DPN subjects (without DFU history), and 10 DPN + DFU subjects. The results showed that patients with diabetes group had significantly higher plantar pressure compared to the HO and HY groups, and the midfoot PTI was significantly higher in the DPN + DFU group compared to the other two groups. In a cross-sectional study by McKay et al. (29), plantar pressure distribution was tested and analyzed in 1000 healthy subjects ranging from 3 to 101 years of age using a plantar force plate. The study found that the plantar PP gradually increased from childhood to the elderly population. PP in children was mainly concentrated in the forefoot region, while in adolescents and the elderly, it was more concentrated in the rearfoot region. Among the elderly, the PTI was higher in the forefoot, midfoot, and the entire foot compared to other age groups.

In essence, an expansive body of research has delved into plantar pressure distribution in the elderly and diabetic populations, providing valuable insights into potential mechanisms that may contribute to the development of foot disorders and the design of mechanical intervention tools such as footwear and orthoses. However, investigations into plantar shear stress have been less extensive. This limitation could stem from difficulties in securing consistent and dependable sensor-based measurements or a historical oversight regarding the significance of shear forces in research contexts. As a result, the findings remain inconsistent, and a definitive comprehension of plantar shear stress—akin to our grasp of plantar pressure distribution—remains elusive (30–32). Furthermore, most of the research related to plantar shear stress testing has focused on sensor design and system development. As described in a systematic review and meta-analysis by Jones et al. (33) in 2021, there is considerable methodological and technical heterogeneity in the research on plantar shear stress testing in diabetic foot. Advancements in sensor technology are imperative before widespread clinical measurements can be implemented, and the focus is gradually shifting towards wearable devices. In recent years, a substantial body of evidence has demonstrated the significant clinical relevance of shear stress to foot ulceration (34, 35). Delbridge et al. (35) pointed out that shear stress, compared to plantar pressure, is more likely to cause rupture of the subcutaneous soft tissue and lead to ulceration. Shear stress acts horizontally in the AP and ML directions at the foot-ground interface, transmitting a complex stress-strain pattern to the deep layers of the plantar soft tissue. These alternating stresses exert abrasive effects on the plantar soft tissue, particularly during the walking process. Just like a running chainsaw, the cutting force produced by the rotating chain easily cuts through a tree trunk, whereas without this shear force, it would be unlikely for the chainsaw to cut through the same trunk by applying vertical pressure alone when the engine is off. Shear stress not only causes surface abrasion of the skin but also damages deeper tissue structures and contributes to the frequent development of calluses in diabetic or elderly feet, which is a well-known risk factor for ulceration. In individuals with diabetes, due to the neuropathy and vascular complications characteristic, patients often experience diminished sensation and compromised blood supply to the feet, impairing their ability to accommodate plantar shear forces. When the plantar aspect is subjected to excessive shear forces, it can lead to skin and deeper soft tissue damage, precipitating the formation of ulcers. Similarly, in elderly populations, plantar shear forces are closely linked to lower extremity conditions. With aging, there is a gradual decline in muscle strength and elasticity, along with a thinning of the plantar fat pad, which diminishes the capacity to resist shear forces. Additionally, the prevalence of diseases such as osteoporosis and arthritis is higher in older adults, further affecting the ability to withstand shear forces. Excessive plantar shear forces can result in fractures, sprains, and other injuries to the foot, exacerbating symptoms of age-related lower extremity disorders

Currently available approaches for the prevention or treatment of DFUs include the following categories: total contact casts, removable casts/braces, therapeutic shoes/boots, ankle-foot orthoses, and therapeutic insoles. Among these, therapeutic insoles are currently a hot research topic due to their lightweight design, excellent compliance, cost-effectiveness, and ability to redistribute and/or cushion shear stress on the plantar surface, thereby reducing the risk of ulcers. A multitude of products on the market possess the functionality of preventing DFU risk and have been proven effective in reducing plantar pressure and the risk of ulcer formation/recurrence through clinical trials. Due to their relatively simple design, validation of the pressure cushioning effect is convenient, and related technical methods are quite mature, resulting in a wealth of research findings and related converted products readily available for use. Conversely, attention to plantar shear stress has only gradually increased in recent years, with fewer preliminary studies conducted, more complex technological approaches, and intricate designs required for shear force cushioning insoles, making such research relatively rare and the number of converted products sparse. Our systematic literature search revealed that only five English-language publications (36–40) report on cushioned insoles for shear forces, and the products mentioned in these five articles are limited to just two types: GlideSoft (36–38) and dynamic foot orthoses (DFO) (39, 40). In this study, in addition to monitoring plantar pressure, we paid special attention to AP and ML shear forces. We found significant changes in the distribution of shear forces in both static and gait conditions in diabetic and HO populations, which corroborates and explains why only 38% of DFU locations match the peak plantar pressure positions reported in the literature (12), and why 41% of patients still experience recurrent plantar calluses after professional plantar decompression therapy (13). This strong correlation with neglecting shear forces underscores the importance of considering both plantar pressure reduction and shear force mitigation in the design of future insoles or other plantar cushioning devices.

Integrating the existing evidence, it is evident that when examining plantar soft tissue in diabetic and geriatric populations, concurrent evaluation of both vertical loads and horizontal shear forces is essential to enhance the prediction accuracy of DFU risks or foot discomfort in the elderly. Future research should further explore the clinical significance of shear stress in large longitudinal cohort studies, providing new references for clinicians and engineers to identify emerging ulcers and foot pain and design effective prevention methods and devices. This study designed a cross-sectional observational study to test the plantar mechanics distribution using static and dynamic experiments. The results showed that the mechanical distributions in the three dimensions were significantly higher in both diabetes patients and HO adults Similarly, compared to HO adults, diabetes patients showed a significant increase in AP shear FTI during gait cycles. Across the seven foot regions, the most pronounced escalation in three-dimensional stress occurred under the M_2-3_ and the heel regions. For measuring the distribution of AP and ML static shear forces, this study used a six-degree-of-freedom perturbation platform that tilted the force plate to a 5° slope. Participants stood statically on the slope to apply a stable and uniform horizontal shear force to the foot. The choice of a 5° slope angle was based on two main reasons: (i) Firstly, preliminary surveys indicated that the majority of participants felt psychologically at ease and could proficiently complete the test on this gradient, minimizing psychological interference and ensuring the safety of the testing procedure. (ii) Secondly, some preliminary data indicated that the static standing on a 5° slope resulted in shear forces on the foot that were close to the magnitude of horizontal shear forces during gait cycles (see data in **Supplementary Tables 4**, **6**).

This study is subject to several limitations. Firstly, the height of the perturbation platform (approximately 1.2 meters) posed a challenge, necessitating the exclusion of older adults over 70 years with compromised balance to safeguard their safety during the experiment. Choosing 70 as the cutoff was based on statistical analysis of balance capabilities and fall risk in the preliminary experiment stage. Additionally, we have considered the representativeness of the study population. Including participants aged 50 to 70 allows for a broad examination of the effects of aging on balance and response to perturbations, while still managing the safety risks associated with the experimental setup. Thus, the age range was narrowed to 50-70 years, encompassing middle-aged and older adults. Secondly, this study was a cross-sectional observational study, and therefore, long-term follow-up was not conducted. It was not possible to observe the long-term pathological changes in foot skin and soft tissues (such as callus formation, diabetic foot ulcers, foot pain, etc.) in each group. Consequently, interplay between the changed plantar mechanics and the progression of related pathologies remains unascertained. It is recommended that future research incorporate large-scale, long-term, prospective observational studies to further elucidate the potential relationship between various foot mechanical indicators and the development of foot diseases in both diabetic and aging populations.

## 5 Conclusions

In summary, diabetic patients and HO individuals exhibited a significant increase in static and dynamic pressure and shear forces on the plantar regions, compared to HY individuals. Notably, the M_2-3_ and the heel regions displayed the most pronounced increases in mechanical loading, underscoring the imperative to prioritize these areas in the design and development of plantar cushioning devices, footwear, or insoles. During a single gait cycle, the time cumulative effect of plantar stress was significantly increased in diabetic and HO populations. Furthermore, this study reaffirms the pivotal role of horizontal shear forces in age-related and diabetic foot deformities. Accordingly, when developing and designing foot cushioning devices, it is it is paramount to integrate strategies that optimize cushioning along the horizontal shear plane, complementing vertical pressure alleviation, to deliver the utmost protective benefits against the development of DFUs and age-related foot discomfort.

## Data Availability

The raw data supporting the conclusions of this article will be made available by the authors, without undue reservation.

## Supplementary material

Supplementary Figure 1 Experimental diagram illustrating the dynamic 3D mechanical distribution of the plantar foot during the gait cycle. The subjects performed a “two-step method” with a natural gait, walking forward from the starting point. The force plate captured the corresponding data during the middle step. The same steps were performed for both the left and right feet.

Supplementary Figure 2 Principal component analysis (PCA) and automatic partitioning of the footprint. PCA was applied to identify the primary axis direction of the footprint, and the footprint was automatically partitioned into seven different rectangular regions based on predefined ratios.

## References

[1] Saeedi P Petersohn I Salpea P Malanda B Karuranga S Unwin N. Global and regional diabetes prevalence estimates for 2019 and projections for 2030 and 2045: Results from the International Diabetes Federation Diabetes Atlas, 9th edition. Diabetes Res Clin Pract. (2019) 157:107843. doi: 10.1016/j.diabres.2019.107843 31518657

[2] Li Y Teng D Shi X Qin G Qin Y Quan H. Prevalence of diabetes recorded in mainland China using 2018 diagnostic criteria from the American Diabetes Association: national cross sectional study. BMJ. (2020) 369:m997. doi: 10.1136/bmj.m997 32345662 PMC7186854

[3] Singh N Armstrong DG Lipsky BA. Preventing foot ulcers in patients with diabetes. JAMA. (2005) 293:217–28. doi: 10.1001/jama.293.2.217 15644549

[4] Armstrong DG Swerdlow MA Armstrong AA Conte MS Padula WV Bus SA. Five year mortality and direct costs of care for people with diabetic foot complications are comparable to cancer. J Foot Ankle Res. (2020) 13:16. doi: 10.1186/s13047-020-00383-2 32209136 PMC7092527

[5] Kerr M Barron E Chadwick P Evans T Kong WM Rayman G. The cost of diabetic foot ulcers and amputations to the National Health Service in England. Diabetes Med. (2019) 36:995–1002. doi: 10.1111/dme.13973 31004370

[6] Boulton AJ. The diabetic foot. Medicine. (2015) 43:33e7. doi: 10.1016/j.mpmed.2014.10.006

[7] Arosi I Hiner G Rajbhandari S. Pathogenesis and treatment of callus in the diabetic foot. Curr Diabetes Rev. (2016) 12(3):179–83. doi: 10.2174/1573399811666150609160219 26054651

[8] Lavery LA Peters EJ Armstrong DG. What are the most effective interventions in preventing diabetic foot ulcers? Int Wound J. (2008) 5:425–33. doi: 10.1111/j.1742-481X.2007.00378.x PMC7951312 18593392

[9] Duffin AC Kidd R Chan A Donaghue KC. High plantar pressure and callus in diabetic adolescents. Incidence and treatment. J Am Podiatr Med Assoc. (2003) 93:214–20. doi: 10.7547/87507315-93-3-214 12756312

[10] Menz HB Zammit GV Munteanu SE. Plantar pressures are higher under callused regions of the foot in older people. Clin Exp Dermatol. (2007) 32:375–80. doi: 10.1111/j.1365-2230.2007.02421.x 17425648

[11] Bus SA Armstrong DG Crews RT Gooday C Jarl G Kirketerp-Moller K. Guidelines on offloading foot ulcers in persons with diabetes (IWGDF 2023 update). Diabetes Metab Res Rev. (2024) 40:e3647. doi: 10.1002/dmrr.v40.3 37226568

[12] Veves A Murray HJ Young MJ Boulton AJ. The risk of foot ulceration in diabetic patients with high foot pressure: a prospective study. Diabetologia. (1992) 35:660–3. doi: 10.1007/BF00400259 1644245

[13] Scirè V Leporati E Teobaldi I Nobili LA Rizzo L Piaggesi A. Effectiveness and safety of using Podikon digital silicone padding in the primary prevention of neuropathic lesions in the forefoot of diabetic patients. JAPMA. (2009) 99:28–34. doi: 10.7547/0980028 19141719

[14] Rubin L. Hyperkeratosis in response to mechanical irritation. J Invest Dermatol. (1949) 13:313–15. doi: 10.1038/jid.1949.102 15398758

[15] Thomas SE Dykes PJ Marks R. Plantar hyperkeratosis. a study of callosities and normal plantar skin. J Invest Dermatol. (1985) 85:394–7. doi: 10.1111/1523-1747.ep12277052 2932504

[16] Menz HB. Biomechanics of the ageing foot and ankle: A mini-review. Gerontology. (2015) 61:381–8. doi: 10.1159/000368357 25402236

[17] Dunn JE Link CL Felson DT Crincoli MG Keysor JJ McKinlay JB. Prevalence of foot and ankle conditions in a multiethnic community sample of older adults. Am J Epidemiol. (2004) 159:491–8. doi: 10.1093/aje/kwh071 14977645

[18] Black JR Hale WE. Prevalence of foot complaints in the elderly. J Am Podiatr Med Assoc. (1987) 77:308–11. doi: 10.7547/87507315-77-6-308 3612508

[19] Murray HJ Young MJ Hollis S Boulton AJ. The association between callus formation, high pressures and neuropathy in diabetic foot ulceration. Diabetes Med. (1996) 13:979–82. doi: 10.1002/(SICI)1096-9136(199611)13:11<979::AID-DIA267>3.0.CO;2-A 8946157

[20] Deng H Li B Shen Q Zhang C Kuang L Chen R. Mechanisms of diabetic foot ulceration: A review. J Diabetes. (2023) 15(4):299–312. doi: 10.1111/1753-0407.13372 PMC10101842 36891783

[21] Chinese Diabetes Society. Guideline for the prevention and treatment of type 2 diabetes mellitus in China (2020 edition). Chin J Diabetes Mellitus. (2021) 13:317–411. doi: 10.3760/cma.j.cn115791-20210221-00095

[22] Qian L Yang X Ma X Yu Y Chen WM. Integration of reginal shear measurements at the foot-ground interface during routine balance assessment of the elderly population. Gait Posture. (2022) 96:18–21. doi: 10.1016/j.gaitpost.2022.05.008 35550502

[23] Hu XX Yang XG Wang X Ma X Geng X. The influence of diabetes and age-related degeneration on body balance control during static standing: a study based on plantar center-of-pressure trajectories and principal component analysis. J Orthop Surg Res. (2023) 18:740. doi: 10.1186/s13018-023-04129-1 37775789 PMC10542244

[24] Rajala S Lekkala J. Plantar shear stress measurements - A review. Clin Biomech (Bristol Avon). (2014) 29:475–83. doi: 10.1016/j.clinbiomech.2014.04.009 24820135

[25] Davis A Pemberton T Ghosh S Maffulli N Padhiar N. Plantar pressure of clipless and toe-clipped pedals in cyclists - A pilot study. Muscles Ligaments Tendons J. (2011) 1:20–4. PMC3666464 23738240

[26] Wang L Jones D Chapman GJ Siddle HJ Russell DA Alazmani A. A review of wearable sensor systems to monitor plantar loading in the assessment of diabetic foot ulcers. IEEE Trans BioMed Eng. (2020) 67:1989–2004. doi: 10.1109/TBME.2019.2953630 31899409

[27] Mueller MJ Zou D Bohnert KL Tuttle LJ Sinacore DR. Plantar stresses on the neuropathic foot during barefoot walking. Phys Ther. (2008) 88:1375–84. doi: 10.2522/ptj.20080011 PMC2579907 18801862

[28] Bacarin TA Sacco IC Hennig EM. Plantar pressure distribution patterns during gait in diabetic neuropathy patients with a history of foot ulcers. Clinics (Sao Paulo). (2009) 64:113–20. doi: 10.1590/s1807-59322009000200008 PMC2666475 19219316

[29] Scott G Menz HB Newcombe L. Age-related differences in foot structure and function. Gait Posture. (2007) 26:68–75. doi: 10.1016/j.gaitpost.2006.07.009 16945538

[30] Yavuz M Master H Garrett A Lavery LA Adams LS. Peak plantar shear and pressure and foot ulcer locations: a call to revisit ulceration pathomechanics. Diabetes Care. (2015) 38:e184–5. doi: 10.2337/dc15-1596 PMC4613917 26370381

[31] Yavuz M Brem RW Glaros AG Garrett A Flyzik M Lavery L. Association between plantar temperatures and triaxial stresses in individuals with diabetes. Diabetes Care. (2015) 38:e178–9. doi: 10.2337/dc15-1147 PMC4613915 26316629

[32] Du L Zhu X Zhe J. An inductive sensor for real-time measurement of plantar normal and shear forces distribution. IEEE Trans BioMed Eng. (2015) 62:1316–23. doi: 10.1109/TBME.2014.2386136 25546856

[33] Jones AD De Siqueira J Nixon JE Siddle HJ Culmer PR Russell DA. Plantar shear stress in the diabetic foot: A systematic review and meta-analysis. Diabetes Med. (2022) 39:e14661. doi: 10.1111/dme.14661 34324731

[34] Brand PW. Tenderizing the foot. Foot Ankle Int. (2003) 24:457–61. doi: 10.1177/107110070302400602 12854665

[35] Delbridge L Ctercteko G Fowler C Reeve TS Le Quesne LP. The aetiology of diabetic neuropathic ulceration of the foot. Br J Surg. (1985) 72:1–6. doi: 10.1002/bjs.1800720102 3881153

[36] Lavery LA LaFontaine J Higgins KR Lanctot DR Constantinides G. Shear-reducing insoles to prevent foot ulceration in high-risk diabetic patients. Adv Skin Wound Care. (2012) 25:519–24. doi: 10.1097/01.ASW.0000422625.17407.93 23080240

[37] Lavery LA Lanctot DR Constantinides G Zamorano RG Athanasiou KA Agrawal CM. Wear and biomechanical characteristics of a novel shear-reducing insole with implications for high-risk persons with diabetes. Diabetes Technol Ther. (2005) 7:638–46. doi: 10.1089/dia.2005.7.638 16120040

[38] Lavery LA Higgins KR La Fontaine J Zamorano RG Constantinides GP Kim PJ. Randomised clinical trial to compare total contact casts, healing sandals and a shear-reducing removable boot to heal diabetic foot ulcers. Int Wound J. (2015) 12:710–5. doi: 10.1111/iwj.12213 PMC7950500 24618113

[39] Belmont B Wang Y Ammanath P Wrobel JS Shih A. An apparatus to quantify anteroposterior and mediolateral shear reduction in shoe insoles. J Diabetes Sci Technol. (2013) 7:410–9. doi: 10.1177/193229681300700218 PMC3737643 23567000

[40] Wrobel JS Ammanath P Le T Luring C Wensman J Grewal GS. A novel shear reduction insole effect on the thermal response to walking stress, balance, and gait. J Diabetes Sci Technol. (2014) 8:1151–6. doi: 10.1177/1932296814546528 PMC4455476 25107709

