# Supplementary figures and images for: The correlation between Diabetes and age-related degeneration and the static and dynamic 3D mechanical distribution of different plantar regions
Source: Front Endocrinol (Lausanne). 2024 Nov 25;15:1433928. doi: 10.3389/fendo.2024.1433928 (PMC11629148; doi:10.3389/fendo.2024.1433928)

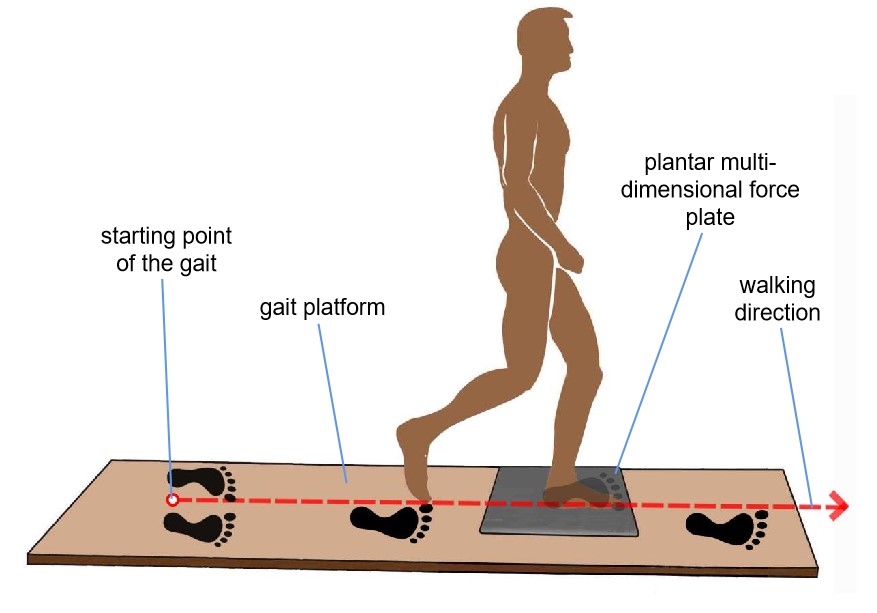

Supplement: Supplementary Figure 1 — Experimental diagram illustrating the dynamic 3D mechanical distribution of the plantar foot during the gait cycle. The subjects performed a “two-step method” with a natural gait, walking forward from the starting point. The force plate captured the corresponding data during the middle step. The same steps were performed for both the left and right feet. [file Image1.jpeg]

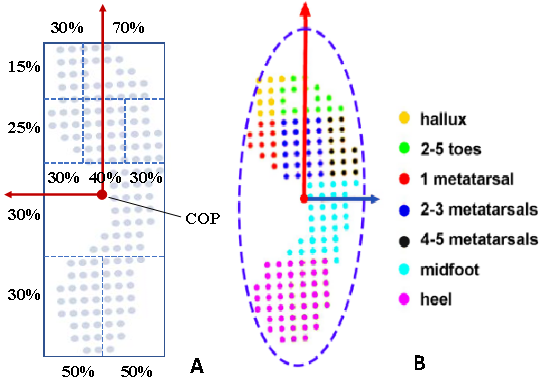

Supplement: Supplementary Figure 2 — Principal component analysis (PCA) and automatic partitioning of the footprint. PCA was applied to identify the primary axis direction of the footprint, and the footprint was automatically partitioned into seven different rectangular regions based on predefined ratios. [file Image2.jpeg]
