# Supplementary material for: The correlation between Diabetes and age-related degeneration and the static and dynamic 3D mechanical distribution of different plantar regions
Source: Front Endocrinol (Lausanne). 2024 Nov 25;15:1433928. doi: 10.3389/fendo.2024.1433928 (PMC11629148; doi:10.3389/fendo.2024.1433928)
Supplement: Supplementary file 4 [file Table1.docx]

| **Supplementary Table S1.** Comparison of the pressure-time integral of different plantar regions during the gait cycle | | | | | | | |
| --- | --- | --- | --- | --- | --- | --- | --- |
| **Regions** | **Group A (N·s)** | **Group B (N·s)** | **Group C (N·s)** | **P value (overall)** | **P value (A vs. B)** | **P value (A vs. C)** | **P value (B vs. C)** |
| entire plantar | 575.53±130.25 | 576.3±128.71 | 675.28±106.69 | 0.003^H**^ | 0.004** | 0.007** | 0.999 |
| hallux | 32.58±15.73 | 41±26.32 | 36.01±15.45 | 0.545^H^ | 0.994 | 0.594 | 0.539 |
| T_2-5_ | 15.68±10.56 | 24.45±15.46 | 14.83±8.10 | 0.012^H*^ | 0.037* | 0.999 | 0.018* |
| M_1_ | 46.17±17.29 | 52.59±24.53 | 58.69±18.28 | 0.069^F^ | 0.208 | 0.045* | 0.248 |
| M_2-3_ | 120.01±36.47 | 110.41±36.75 | 170.34±32.82 | <0.001^H***^ | <0.001*** | <0.001*** | 0.468 |
| M_4-5_ | 59.27±31.66 | 50.21±32.77 | 64.49±19.05 | 0.061^H^ | 0.044* | 0.216 | 0.469 |
| LA | 95.24±43.19 | 90.05±45.68 | 90.86±34.68 | 0.821^H^ | 0.862 | 0.963 | 0.796 |
| heel | 208.36±55.64 | 207.02±63.55 | 229.62±65.07 | 0.356^F^ | 0.365 | 0.204 | 0.936 |

**Footnotes**: Group A: healthy younger subjects; group B: healthy older subjects; group C: patients with diabetes. F and H represent the effect sizes of one-way ANOVA and Kruskal-Wallis H test, respectively. SNK-q test and Dunnett's test were used for *post-hoc* multiple comparisons corresponding to the two statistical analyses. The data are presented as “mean±SD”. T_2-5_: 2^nd^-5^th^ toes; M_1_, 1^st^ metatarsal head; M_2-3_, 2^nd^-3^rd^ metatarsal heads; M_4-5_, 4^th^-5^th^ metatarsal heads; LA, lateral arch region.*P<0.05,**P<0.01, ***P<0.001.
