# Supplementary material for: The correlation between Diabetes and age-related degeneration and the static and dynamic 3D mechanical distribution of different plantar regions
Source: Front Endocrinol (Lausanne). 2024 Nov 25;15:1433928. doi: 10.3389/fendo.2024.1433928 (PMC11629148; doi:10.3389/fendo.2024.1433928)
Supplement: Supplementary file 5 [file Table2.docx]

| **Supplementary Table S2.** Comparison of the peak pressure of different plantar regions during the gait cycle | | | | | | | |
| --- | --- | --- | --- | --- | --- | --- | --- |
| **Regions** | **Group A(N)** | **Group B(N)** | **Group C(N)** | **P value (overall)** | **P value (A vs. B)** | **P value (A vs. C)** | **P value (B vs. C)** |
| entire plantar | 624.69±102.37 | 595.45±77.34 | 698.26±81.33 | <0.001^H***^ | 0.002** | <0.001*** | 0.343 |
| hallux | 47.31±19.56 | 53.54±34.97 | 54.89±25.12 | 0.701^H^ | 0.631 | 0.763 | 0.970 |
| T_2-5_ | 26.90±15.43 | 37.97±21.95 | 23.11±10.49 | 0.006^H**^ | 0.675 | 0.007** | 0.036* |
| M_1_ | 65.08±20.57 | 71.67±30.82 | 84.15±24.13 | 0.035^F*^ | 0.021* | 0.078 | 0.349 |
| M_2-3_ | 163.93±42.84 | 138.48±49.05 | 227.40±41.29 | <0.001^H***^ | <0.001*** | <0.001*** | 0.037 |
| M_4-5_ | 87.28±37.39 | 70.80±41.19 | 91.61±25.94 | 0.029^H*^ | 0.528 | 0.026* | 0.142 |
| LA | 136.90±48.05 | 126.64±49.11 | 138.70±36.92 | 0.322^H^ | 0.792 | 0.224 | 0.605 |
| heel | 299.77±68.59 | 278.68±55.37 | 346.21±78.16 | 0.002^H**^ | 0.049* | 0.003** | 0.515 |

**Footnotes**: Group A: healthy younger subjects; group B: healthy older subjects; group C: patients with diabetes. F and H represent the effect sizes of one-way ANOVA and Kruskal-Wallis H test, respectively. SNK-q test and Dunnett's test were used for *post-hoc* multiple comparisons corresponding to the two statistical analyses. The data are presented as “mean±SD”. T_2-5_: 2^nd^-5^th^ toes; M_1_, 1^st^ metatarsal head; M_2-3_, 2^nd^-3^rd^ metatarsal heads; M_4-5_, 4^th^-5^th^ metatarsal heads; LA, lateral arch region.*P<0.05,**P<0.01, ***P<0.001.
