# Supplementary material for: The correlation between Diabetes and age-related degeneration and the static and dynamic 3D mechanical distribution of different plantar regions
Source: Front Endocrinol (Lausanne). 2024 Nov 25;15:1433928. doi: 10.3389/fendo.2024.1433928 (PMC11629148; doi:10.3389/fendo.2024.1433928)
Supplement: Supplementary file 6 [file Table3.docx]

| **Supplementary Table S3.** Comparison of the anterior-posterior shear force-time integral of different plantar regions during the gait cycle | | | | | | | |
| --- | --- | --- | --- | --- | --- | --- | --- |
| **Regions** | **Group A (N·s)** | **Group B (N·s)** | **Group C (N·s)** | **P value (overall)** | **P value (A vs. B)** | **P value (A vs. C)** | **P value (B vs. C)** |
| entire plantar | 28.42±6.13 | 29.62±6.45 | 31.85±5.03 | 0.022^F*^ | 0.828 | 0.028* | 0.042* |
| hallux | 1.51±0.86 | 1.93±1.43 | 1.59±0.79 | 0.403^H^ | 0.520 | 0.948 | 0.363 |
| T_2-5_ | 0.73±0.55 | 1.19±0.86 | 0.62±0.38 | 0.002^H**^ | 0.003** | 0.695 | 0.014* |
| M_1_ | 2.32±0.92 | 2.76±1.17 | 2.43±0.75 | 0.011^BF*^ | 0.040* | 0.987 | 0.016* |
| M_2-3_ | 5.8±2.12 | 5.64±2.19 | 7.28±1.34 | 0.015^F*^ | 0.021* | 0.011* | 0.533 |
| M_4-5_ | 2.62±1.48 | 2.31±1.50 | 2.64±0.81 | 0.709^BF^ | 0.539 | 0.942 | 0.770 |
| LA | 4.07±1.86 | 4.03±1.94 | 3.64±1.54 | 0.288^F^ | 0.241 | 0.317 | 0.538 |
| heel | 10.85±2.99 | 11.19±2.75 | 12.68±3.13 | 0.047^H*^ | 0.667 | 0.046* | 0.159 |

**Footnotes**: Group A: healthy younger subjects; group B: healthy older subjects; group C: patients with diabetes. F, H and BF represent the effect sizes of one-way ANOVA, Kruskal-Wallis H test, and Brown-Forsythe test, respectively. SNK-q test, Dunnett's test and Tamhane’s T_2_ were used for *post-hoc* multiple comparisons corresponding to the three statistical analyses. The data are presented as “mean±SD”. T_2-5_: 2^nd^-5^th^ toes; M_1_, 1^st^ metatarsal head; M_2-3_, 2^nd^-3^rd^ metatarsal heads; M_4-5_, 4^th^-5^th^ metatarsal heads; LA, lateral arch region.*P<0.05,**P<0.01, ***P<0.001.
