# Supplementary material for: The correlation between Diabetes and age-related degeneration and the static and dynamic 3D mechanical distribution of different plantar regions
Source: Front Endocrinol (Lausanne). 2024 Nov 25;15:1433928. doi: 10.3389/fendo.2024.1433928 (PMC11629148; doi:10.3389/fendo.2024.1433928)
Supplement: Supplementary file 7 [file Table4.docx]

| **Supplementary Table S4.** Comparison of the anterior-posterior peak shear forces of different plantar regions during the gait cycle | | | | | | | |
| --- | --- | --- | --- | --- | --- | --- | --- |
| **Regions** | **Group A(N)** | **Group B(N)** | **Group C(N)** | **P value (overall)** | **P value (A vs. B)** | **P value (A vs. C)** | **P value (B vs. C)** |
| entire plantar | 49.28±9.26 | 46.19±7.12 | 48.57±7.64 | 0.708^F^ | 0.469 | 0.708 | 0.947 |
| hallux | 3.34±1.34 | 3.35±2.23 | 3.99±2.82 | 0.588^F^ | 0.349 | 0.609 | 0.991 |
| T_2-5_ | 1.99±1.21 | 2.73±2.03 | 1.39±0.84 | 0.004^H**^ | 0.004** | 0.071 | 0.200 |
| M_1_ | 4.60±1.97 | 6.69±10.64 | 4.00±1.47 | 0.107^H^ | 0.061 | 0.352 | 0.638 |
| M_2-3_ | 10.16±2.9 | 9.04±3.66 | 11.10±2.38 | 0.278^F^ | 0.241 | 0.470 | 0.372 |
| M_4-5_ | 5.36±2.61 | 4.09±2.53 | 4.75±1.53 | 0.176^F^ | 0.466 | 0.305 | 0.188 |
| LA | 7.66±3.58 | 7.19±2.98 | 6.51±2.08 | 0.297^H^ | 0.243 | 0.328 | 0.935 |
| heel | 22.15±5.24 | 21.46±3.52 | 24.24±6.39 | 0.420^F^ | 0.523 | 0.367 | 0.475 |

**Footnotes**: Group A: healthy younger subjects; group B: healthy older subjects; group C: patients with diabetes. F and H represent the effect sizes of one-way ANOVA and Kruskal-Wallis H test, respectively. SNK-q test and Dunnett's test were used for *post-hoc* multiple comparisons corresponding to the two statistical analyses. The data are presented as “mean±SD”. T_2-5_: 2^nd^-5^th^ toes; M_1_, 1^st^ metatarsal head; M_2-3_, 2^nd^-3^rd^ metatarsal heads; M_4-5_, 4^th^-5^th^ metatarsal heads; LA, lateral arch region. *P<0.05, **P<0.01, ***P<0.001.
