# Supplementary material for: The correlation between Diabetes and age-related degeneration and the static and dynamic 3D mechanical distribution of different plantar regions
Source: Front Endocrinol (Lausanne). 2024 Nov 25;15:1433928. doi: 10.3389/fendo.2024.1433928 (PMC11629148; doi:10.3389/fendo.2024.1433928)
Supplement: Supplementary file 8 [file Table5.docx]

| **Supplementary Table S5.** Comparison of the medial-lateral shear force-time integral of different plantar regions during the gait cycle | | | | | | | |
| --- | --- | --- | --- | --- | --- | --- | --- |
| **Regions** | **Group A (N·s)** | **Group B (N·s)** | **Group C (N·s)** | **P value (overall)** | **P value (A vs. B)** | **P value (A vs. C)** | **P value (B vs. C)** |
| entire plantar | 20.64±13.47 | 22.9±12.95 | 23.24±15.17 | 0.257^H^ | 0.972 | 0.422 | 0.229 |
| hallux | 1.26±1.05 | 1.47±1.32 | 1.13±0.65 | 0.598^H^ | 0.608 | 0.998 | 0.580 |
| T_2-5_ | 0.68±0.78 | 0.97±0.86 | 0.53±0.40 | 0.033^H*^ | 0.029* | 0.787 | 0.105 |
| M_1_ | 1.67±1.17 | 2.02±1.48 | 1.96±1.63 | 0.563^H^ | 0.725 | 0.910 | 0.524 |
| M_2-3_ | 4.34±2.90 | 4.20±2.93 | 5.64±3.91 | 0.336^H^ | 0.537 | 0.265 | 0.971 |
| M_4-5_ | 2.00±1.58 | 1.70±1.31 | 2.11±1.52 | 0.801^H^ | 0.815 | 0.894 | 0.870 |
| LA | 3.15±2.85 | 3.24±2.56 | 3.06±2.57 | 0.545^H^ | 0.548 | 0.900 | 0.690 |
| heel | 7.01±4.85 | 8.05±5.13 | 8.30±5.74 | 0.331^H^ | 0.868 | 0.564 | 0.860 |

**Footnotes**: Group A: healthy younger subjects; group B: healthy older subjects; group C: patients with diabetes. H represents the effect size of Kruskal-Wallis H test, and the Dunnett's test was used for *post-hoc* multiple comparisons. The data are presented as “mean±SD”. T_2-5_: 2^nd^-5^th^ toes; M_1_, 1^st^ metatarsal head; M_2-3_, 2^nd^-3^rd^ metatarsal heads; M_4-5_, 4^th^-5^th^ metatarsal heads; LA, lateral arch region.*P<0.05, **P<0.01, ***P<0.001.
