# Supplementary material for: The correlation between Diabetes and age-related degeneration and the static and dynamic 3D mechanical distribution of different plantar regions
Source: Front Endocrinol (Lausanne). 2024 Nov 25;15:1433928. doi: 10.3389/fendo.2024.1433928 (PMC11629148; doi:10.3389/fendo.2024.1433928)
Supplement: Supplementary file 9 [file Table6.docx]

| **Supplementary Table S6.** Comparison of the medial-lateral peak shear forces of different plantar regions during the gait cycle | | | | | | | |
| --- | --- | --- | --- | --- | --- | --- | --- |
| **Regions** | **Group A(N)** | **Group B(N)** | **Group C(N)** | **P value (overall)** | **P value (A vs. B)** | **P value (A vs. C)** | **P value (B vs. C)** |
| entire plantar | 33.84±14.02 | 34.43±18.23 | 33.01±12.67 | 0.830^H^ | 0.766 | 0.300 | 0.940 |
| hallux | 2.39±1.51 | 2.30±1.47 | 2.32±0.85 | 0.950^H^ | 0.970 | 0.946 | 0.961 |
| T_2-5_ | 1.59±1.37 | 1.88±1.37 | 1.10±0.68 | 0.068^H^ | 0.040* | 0.239 | 0.515 |
| M_1_ | 3.20±1.71 | 5.32±11.92 | 3.10±2.35 | 0.501^H^ | 0.402 | 0.767 | 0.906 |
| M_2-3_ | 8.15±5.28 | 5.94±3.84 | 8.66±4.90 | 0.206^H^ | 0.254 | 0.770 | 0.312 |
| M_4-5_ | 3.54±2.39 | 2.49±1.63 | 3.41±2.04 | 0.541^H^ | 0.557 | 0.557 | 1.000 |
| LA | 5.62±4.00 | 5.35±3.59 | 5.00±3.59 | 0.648^H^ | 0.660 | 0.837 | 0.812 |
| heel | 12.16±6.60 | 13.00±7.16 | 14.71±7.39 | 0.381^H^ | 0.816 | 0.330 | 0.556 |

**Footnotes**: Group A: healthy younger subjects; group B: healthy older subjects; group C: patients with diabetes. H represents the effect size of Kruskal-Wallis H test, and the Dunnett's test was used for *post-hoc* multiple comparisons. The data are presented as “mean±SD”. T_2-5_: 2^nd^-5^th^ toes; M_1_, 1^st^ metatarsal head; M_2-3_, 2^nd^-3^rd^ metatarsal heads; M_4-5_, 4^th^-5^th^ metatarsal heads; LA, lateral arch region.*P<0.05, **P<0.01, ***P<0.001.
